# Synapsin III Regulates Dopaminergic Neuron Development in Vertebrates

**DOI:** 10.3390/cells11233902

**Published:** 2022-12-02

**Authors:** Gaia Faustini, Francesca Longhena, Alessia Muscò, Federica Bono, Edoardo Parrella, Luca La Via, Alessandro Barbon, Marina Pizzi, Franco Onofri, Fabio Benfenati, Cristina Missale, Maurizio Memo, Daniela Zizioli, Arianna Bellucci

**Affiliations:** 1 Department of Molecular and Translational Medicine, University of Brescia, Viale Europa 11, 25123 Brescia, Italy; 2 Department of Experimental Medicine, University of Genova, Via Leon Battista Alberti 2, 16132 Genova, Italy; 3 IRCSS Policlinico San Martino Hospital, Largo Rosanna Benzi 10, 16132 Genova, Italy; 4 Italian Institute of Technology, Via Morego 30, 16163 Genova, Italy; 5 Laboratory for Preventive and Personalized Medicine, Department of Molecular and Translational Medicine, University of Brescia, Viale Europa 11, 25123 Brescia, Italy

**Keywords:** synapsin III, dopaminergic neurons, attention deficit and hyperactivity disorder, neurodevelopment

## Abstract

Attention deficit and hyperactivity disorder (ADHD) is a neurodevelopmental disorder characterized by alterations in the mesocorticolimbic and nigrostriatal dopaminergic pathways. Polymorphisms in the Synapsin III (Syn III) gene can associate with ADHD onset and even affect the therapeutic response to the gold standard ADHD medication, methylphenidate (MPH), a monoamine transporter inhibitor whose efficacy appears related with the stimulation of brain-derived neurotrophic factor (BDNF). Interestingly, we previously showed that MPH can bind Syn III, which can regulate neuronal development. These observations suggest that Syn III polymorphism may impinge on ADHD onset and response to therapy by affecting BDNF-dependent dopaminergic neuron development. Here, by studying zebrafish embryos exposed to Syn III gene knock-down (KD), Syn III knock-out (ko) mice and human induced pluripotent stem cells (iPSCs)-derived neurons subjected to Syn III RNA interference, we found that Syn III governs the earliest stages of dopaminergic neurons development and that this function is conserved in vertebrates. We also observed that in mammals Syn III exerts this function acting upstream of brain-derived neurotrophic factor (BDNF)- and cAMP-dependent protein kinase 5 (Cdk5)-stimulated dendrite development. Collectively, these findings own significant implications for deciphering the biological basis of ADHD.

## 1. Introduction

Synapsin III (Syn III) is a neuronal phosphoprotein regulating striatal dopaminergic neurotransmission in the adult brain [1,2] and is involved in psychiatric disorders associated with dysfunctions of brain dopaminergic systems, including attention deficit and hyperactivity disorder (ADHD) [3,4,5,6]. This latter is characterized by an impaired development of midbrain dopaminergic neurons (mDN), reflected by a marked alteration in brain dopamine (DA) synthesis and dopaminergic dysregulations in the mesocorticolimbic and nigrostriatal pathway [7,8,9].

Interestingly, certain human Syn III gene (SYN3) polymorphisms associate with ADHD onset and influence the response to the most effective ADHD treatment, methylphenidate (MPH), a monoamine reuptake inhibitor which binds Syn III [4,10,11,12]. This hints that Syn III may control the development of mDN.

In this study, we aimed to clarify whether Syn III is actually involved in the regulation of mDN development. To this purpose, we studied zebrafish (*Danio Rerio*) embryos exposed to Syn III gene knock-down (KD), Syn III knockout (ko) mice and human induced pluripotent stem cells (iPSCs)-derived mDN subjected to Syn III RNA interference (RNAi). We found that Syn III governs the very beginnings of dopaminergic neurons development in fish and mammals. Syn III gene KD in zebrafish larvae produced an ADHD-like phenotype and dopaminergic neurodevelopmental delay that was rescued by the expression of rat Syn III mRNA. In mice, Syn III-dependent regulation of mDN development was related with the upstream control of brain-derived neurotrophic factor (BDNF) production and cAMP-dependent protein kinase 5 (Cdk5)-mediated signaling. Our results also support that the early Syn III deletion-dependent mDN developmental deficits observed in mammals can be progressively redeemed, though the recovery of dendritic arborizations is slower than the rescue of neuron number or axonal projections.

## 2. Materials and Methods

### 2.1. Animals

All the experiments on zebrafish were performed with embryos under the age of 5/7 days post-fertilization (dpf), in accordance with the standard rules defined by the Local Commitment for animal Health (Project 211B5-10) and following the Italian and European rules on animal care (EU directive 2010/63/EU). Wild type (wt) AB strain and the transgenic lines Tg(*neurod1*:eGFP) and Tg(*ngn1*:eGFP) were used for all the experiments. Fish were kept in 3 L tanks with 28 °C warm water in a circulating system maintained at pH 7.0–7.5 and conductivity in a range of 450–550 µS. Adult animals were maintained under a 14/10 h light/dark cycle and fed three times per day with a combination of granular food and freshly prepared *Artemia*. Breeding of adult zebrafish was carried out by natural crosses and embryos were collected; they were staged according to established protocols [13]. Fresh spawned embryos produced in the morning were collected in Petri dishes containing fish water (0.1 g/L Instant Ocean Sea Salts, 0.1 g/L Sodium Bicarbonate, 0.19 g/L Calcium Sulfate, 0.2 mg/L methylene blue in reverse osmosis water) until the desired developmental stage was reached and for further experiments. To examine post-gastrulation stages for whole mount in situ hybridization (WISH) experiments, fish water was added by 0.004% 1-phenil-2-thiourea (PTU, P7629, Sigma-Aldrich, St. Louis, MO, USA) solution.

C57BL/6J wt and Syn III ko mice [14,15] were bred at the animal house facility at the Department of Molecular and Translational Medicine of University of Brescia. Animals were maintained under a 12 h light–dark cycle at a room temperature (rt) of 22 °C and had ad libitum food and water. All experiments were made in accordance with the Directive 2010/63/EU of the European Parliament and of the Council of 22 September 2010 on the protection of animals used. All experimental and surgical procedures conformed to the National Research Guide for the Care and Use of Laboratory Animals were approved by the Animal Research Committees of the University of Brescia (Protocol Permit 719/2015-PR). All achievements were made to minimize animal suffering and to reduce the number of animals used. C57BL/6J wt mice (Charles River, Wilmington, MA, USA), and Syn III ko animals on C57BL/6J background [16] were used in this study at 0, 2, and 10 months of age.

### 2.2. Mouse Primary Midbrain Neurons Cultures

Primary midbrain neurons were dissected from C57BL/6J wt control mice and Syn III ko mice at embryonic day 12.5. After dissociation with Accumax (A7089, Sigma-Aldrich), the single cells were re-suspended in whole culture medium composed of Neurobasal^TM^ medium (21103049, Thermo Fisher Scientific, Waltham, MA, USA) containing 100 μg/mL penicillin, 100 μg/mL streptomycin (P4333, Sigma-Aldrich), 2 mM glutamine (ECB3000, EuroClone, Milan, Italy) and 2% B27 supplement (17504044, Thermo Fisher Scientific) and then centrifuged. Cell counts and viability assays were performed using the Trypan Blue exclusion test. Neurons were seeded onto glass coverslides in 24-well plates coated with 12 µg/mL poly-D-lysine. Cells were maintained at 37 °C under a humidified atmosphere of 5% CO_2_ in Neurobasal medium for 1, 3 or 10 days in vitro (div).

### 2.3. Drug Treatments on Mouse Primary Midbrain Neurons Cultures

DA (0.1 µM) diluted in 1 mg/mL ascorbic acid and 10 ng/mL BDNF diluted in PBS/0.1% BSA were used to treat mouse primary midbrain neurons. In particular, DA or BDNF were diluted in whole culture medium that was used to re-suspend cell pellets before seeding. The DA or BDNF supplemented whole culture medium was maintained until div 1, when cells were fixed with Immunofix for 15 min and subjected to morphological analysis. Control cells were re-suspended and cultured in whole culture medium without DA or BDNF addition up to fixation at div 1. The treatment with 1 µM roscovitine (prepared from a 100 mM stock diluted in DMSO) was performed by direct addition of the proper amount of drug solution in the culture medium at div 3 for 24 h. Cells were then re-incubated with standard cell culture media and then fixed at div 10. Control cells were subjected to addition of the same amount of DMSO solution used to perform roscovitine treatment.

### 2.4. Human iPSCs Culture, Transduction and Differentiation into mDN

Two previously described iPSCs control cell lines (CTRL-1 and CTRL-2) that show a complete analysis of pluripotent stem characteristics and derive from adult individuals without any known disease were used in this study. Informed consent was obtained from all healthy donors involved in our study [17,18]. iPSCs were cultured on Matrigel (356234, BD Biosciences, San Jose, CA, USA)-coated 6-well tissue culture plates (Corning, New York, NY, USA) in StemFlex medium (A3349401, Gibco, Thermo Fisher Scientific) changed every other day. Cells were passaged every 3–4 days using ReleSR (05872, Stemcell technologies, Vancouver, BC, Canada) and replated onto fresh Matrigel-coated plates. Cells were cultured at 37 °C in 5% CO_2_ and in a humidified atmosphere.

For SYN3 silencing, iPSCs were infected with an adeno-associated viral vector (AAV) expressing either a short hairpin RNA (shRNA) serotype 2/6 driven by the U6 promoter (Vector biolabs, Malvern, PA, USA) allowing silencing of SYN3 (AAV-shSynIII) or with non-silencing RNA sequences (AAV-shNSC) at 1 × 10^2^ gc/mL for 24 h at 37 °C and 5% CO_2_. After 24 h, the medium containing the viral particles was removed and replaced with fresh medium. SYN3 silencing was checked 6 days post transduction by Western blot (WB) analysis.

Differentiation of iPSCs into mature mDN were obtained using a dual-SMAD inhibition protocol [19] with some modifications, as previously described [17,18]. Cells were then fixed at different time points (div 25, div 40, div 50) during the differentiation process and analyzed by immunocytochemistry for morphological parameters and by WB for SYN3 silencing.

### 2.5. Bioinformatic Analysis of Syn III

The sequence of the genes encoding for Syn III in humans (SYN3), mouse or rat (Syn3) were assembled using ENSEMBL and BLAST searches within the Zebrafish Genome Browser (www.ensembl.org, accessed on 1 September 2022) were used to identify one homologous sequence on chromosome 4 where the zebrafish Syn III gene (*syn3*) is located. For the in silico analysis, we used the following accession numbers: ENSDART000000157825.1 and ZFIN ZDB-GENE141212-325. Nucleotide and amino acid sequences were compared to the non-redundant sequences present at the National Center for Biotechnology Information (NCBI) using the Basic Local Alignment Search Tool (BLAST). Multiple sequences alignment was performed using the CLUSTALW (CLUSTAL 1.8) [20]. The following accession numbers of Syn III genes were used: human (NP_003481.3), mouse (NP_038750.2) and zebrafish (NP_001340893.1). To find evidence for the conservation of synteny, we compared genomic regions neighboring the zebrafish Syn III to the genes neighboring human and mouse synapsins. Putative orthologs for each zebrafish gene were located on the human and mouse map using the Genomicus (http://www.genomicus.biologie.ens.fr/genomicus-100.01/cgi-bin/search.pl, accessed on 1 September 2022) informatic tool.

### 2.6. Morpholino Microinjections and Zebrafish Phenotypic Assessment

To knockdown the expression of Syn III gene, a splice-inhibiting morpholino was used to knockdown *syn3* expression (*Syn III*-MO) (5′-TCGATAACTAAGCGATGCCATCTGT-3′) (GeneTools, LCC) in order to target the intron1-exon1 junction of immature mRNA and produce exon skipping in mRNA maturation by inducing the retention of exon1. A standard non-targeting morpholino (ST-MO) (5′-CCTCTTACCTCAGTTACAATTTATA-3′) was used as control (Gene Tools). We prepared the injection solution by diluting the morpholino stock solution (1 mM) with 10% phenol red and water to obtain the desired concentration to test a dose-curve. The following concentrations of oligo-morpholino were injected into embryos at 1–2 cell stage to determine the optimal concentration: 0.3, 0.5, 1, 1.5 and 2 pmol/embryo. The final concentration of 1 pmol/embryo (corresponding to 8 ng/embryo) was used for all experimental procedures. Four nl of 1 pmol/embryo were injected into 1-2 cell stage embryos by Eppendorf FemtoJet Micromanipulator 5171. Injected embryos were then collected in Petri dishes and maintained in fish water at 28 °C. To avoid pigmentation process, 0.004% PTU was added to fish water at 20 h post-fertilization (hpf) and development and morphological features were evaluated at 24 and 48 hpf. Images were taken using Zeiss AxioZoom V16 equipped with Zeiss Axiocam 503 color digital camera (Carl Zeiss, Jena, Germany) and processed using Zen Pro software (Carl Zeiss). All experiments were repeated at least three times and altogether more than 150 of each type were viewed.

### 2.7. RNA Extraction, Reverse Transcription (RT)-PCR and Quantitative Real Time (qrt)-PCR

Total RNA extraction was performed with the guanidine isothyocianate/phenol method. Frozen pooled embryos (30 embryos/sample) were lysated in TRI reagent^®^ (93289, Sigma-Aldrich, St. Louis, MO, USA) according to manufacturer’s protocol. RNA was isolated using 1-Bromo-3-chloropropane (B62404, Sigma-Aldrich), precipitated in 2-propanol (I9516, Sigma-Aldrich) and resuspended in RNase-free water (AM9916, Thermofisher Scientific). Quantification was performed with mySPEC Microvolume Spectrophotometer (VWR International, Radnor, PA, USA). One microgram RNA was retrotranscribed using QuantiTect Reverse Transcription Kit (205311, Qiagen, Germany). cDNA was used for both RT-PCR and qrt-PCR.

Primers were designed using the Primer-BLAST tool in the National Center for Biotechnology Information (NCBI) browser. The following accession numbers was used as a template for primer pairs search: *Danio rerio* Syn III mRNA: NM_001353964.1.

Primer pairs sequences are listed as follows: *syn3* forward (ccgctaacaagactcagcct); *syn3* reverse (ctgcttctgtgtgtgtgtgc); Rp1l3a forward (ctatgaccaataggaagagcaacc); Rp1l3a reverse (gcagagtatatgaccaggtggaa).

RT-PCR reactions were performed in a 13.5 µL volume, with 0.625 u of GoTaq^®^ DNA Polymerase (Promega), 0.5 µM of each primer and 2 mM MgCl2 in 1X Green GoTaq^®^ Flexi Buffer (Promega) and 50 ng cDNA. cDNA amplification was conducted with the following thermal cycle: 95 °C 2 min initial denaturation, followed by 32 cycles of 50 s 95 °C heat denaturation, 30 s 59 °C annealing, 30 s of 72 °C extension. A supplemental 5 min at 72 °C extension step was carried out at the end. Amplicons were separated on a 1% TBE-agarose gel and DNA bands were revealed with GelRed^®^ Nucleic Acid Stain using an UV Transilluminator.

The qrt-PCR analysis was performed using the Viia7 system (Thermo Fisher Scientific). Reactions were performed in a 10 µL volume, with 2 µM of each primer, Sybr Green MasterMix (1725150, Biorad, Hercules, CA, USA), and 20 ng of cDNA. Amplification profile consisted of a 1-min initial denaturation 95° step, followed by 40 cycles of two-step amplification (at 95 °C for 15 s and at 60 °C for 30 s) and a melting cycle. Each reaction was performed in triplicate and the relative expression of Syn III mRNA was calculated with the ΔΔCt method using rpl13a as reference gene.

### 2.8. WISH

WISH was performed according to a standard protocol [21]. Briefly, embryos were collected, dechorionated and incubated at 28 °C at different stages. They were then fixed for 2 h with 4% paraformaldehyde (PFA, UN22B, Sigma-Aldrich) in PBS at rt, dehydrated through ascending (25%–50%–75%) PBS/methanol solutions (1230, Sigma-Aldrich) and stored at −20 °C. After treatment with 10 μg/mL proteinase K (P2308, Roche, Switzerland), the embryos were hybridized overnight at 68 °C with 1 ng/μL digoxygenin (DIG)-labeled RNA probes kindly provided by prof. Franco Cotelli of the University of Milan (Italy). Embryos were washed with ascending scale of Hybe Wash (50% deionized formamide, 5X SSC buffer, 0.1% Tween 20, adjusted to pH 6.0 by adding citric acid)/PBS 1X and SSC (Sodium-citrate Buffer, Ambion, USA)/PBS then incubated with anti-DIG antibody conjugated with alkaline phosphatase (1:10,000, 11093274910, Roche, Germany) overnight at 4 °C. The staining was performed with NBT/BCIP (blue staining solution, 11681451001, Roche) alkaline phosphatase substrates. WISH images were taken with ZEISS Axio Zoom V.16 microscope (Carl Zeiss) equipped with Zeiss Axiocam 506 color digital camera and processing using Zen Pro software. Quantification of WISH pictures was performed with ImageJ software (NIH, Bethesda, MD, USA) as previously described [22]: a region of interest (ROI) ranging from the top of the head to mid-yolk was selected in 8-bit pictures. Due to the widespread distribution of the colorimetric signal across the embryo structure, background intensity measurement was carried out in blank embryos lacking incubation with RNA probe. The following probes were used: neurogenin-related gene I (*ngn1*); *Islet-1*; glial fibrillary acidic protein gene (*gfap*).

### 2.9. In Vitro Generation of Rat Syn III mRNA Expression Plasmid for Zebrafish Transfection

A recombinant plasmid containing the coding region of rat Syn III mRNA (p3x-FLAG-CMV-14-rat Syn III, a kind gift of Dr. Franco Onofri, University of Genova, Italy) was digested with Xba I/Hind III restriction enzymes and subcloned in pCS2+ plasmid (a kind gift of Prof. Franco Cotelli, University of Milan). The resulting linearized plasmid was transcribed with T7 RNA polymerase using the mMESSAGE mMACHINE T7 Transcription Kit (AM1344, ThermoFisher Scientific, Waltham, MA, USA) according to manufacturer’s protocol. The product was quantified by mySPEC spectrophotometer (VWR International, Milan, Italy) and used for microinjections. A dose-response curve was performed to establish the proper amount of mRNA (100 pg/embryo) to be injected to avoid the appearance of aberrant phenotypes. 100 pg of rat Syn III mRNA was injected alone or co-injected with *Syn III*-MO at 1-2 cell stage embryos.

### 2.10. Acridine Orange Staining on Zebrafish Embryos

To analyze the level of cell death, acridine orange staining was performed using a standard protocol [23]. Embryos at 24 and 48 hpf were dechorionated and incubated for 30 min in fish water containing acridine orange staining solution (10 mg/L). Embryos were then rinsed three times in fish water, mounted using 80% glycerol and quickly imaged using epifluorescent microscope with green fluorescence filter (Zeiss AxioZoom V16 equipped with Zeiss Axiocam 506 color digital camera and processed using Zen Pro software, Carl Zeiss). The acridine orange fluorescence intensity was quantified using ZF-Mapper software.

### 2.11. Immunofluorescence Studies on Zebrafish Embryos

For tyrosine hydroxylase (TH) immunofluorescence, 48 hpf embryos were fixed in 4% PFA for two hours at rt, then rinsed twice in PBS for 1 h. Embryos were permeabilized in 10% methanol in PBS for 1 h under gentle agitation and non-specific binding was blocked overnight in 10% Normal Goat Serum (NGS), 1% BSA in PBS/Triton X-100 0.3%. The embryos were incubated for 72 h with rabbit anti-TH (1:200, ab152, Merck Millipore, Burlington, MA, USA) primary antibody in blocking solution at 4 °C. After three washes in PBS for 1 h, samples were incubated with the anti-rabbit Cy3 (1:100, 111-165-144, Jackson Immunoresearch, Cambridge, UK) secondary antibody diluted in 10% NGS, 1% BSA in PBS for 40 h at 4 °C and then rinsed several times in PBS. TH-stained embryos were visualized using Zeiss LSM880 confocal microscope (Carl Zeiss).

### 2.12. Light-Sheet Imaging

For light-sheet imaging, zebrafish embryos at the desired developmental stage were manually dechorionated and anesthetized in 0.04 mg/L tricaine and mounted in a glass capillary containing 1% low melting agarose (16520050, Thermo Fisher Scientific). After polymerization, capillaries were located into the imaging chamber. Images were acquired with Zeiss Light-sheet V1 (Carl Zeiss) using a 10x detection objective and laser sets at 488 nm. Three-dimensional movies were created using Arivis software (Arivis AG, Munich, Germany).

### 2.13. Confocal Microscopy

For confocal imaging of zebrafish embryos, these were fixed overnight in a PFA-based solution (25% PFA, 0.03 g/mL sucrose, 0.4% glutaraldehyde in PBS), then washed twice in PBS and kept in the same fixative solution with half the PFA, for at least 24 h. The yolk was then removed from the selected embryos to avoid autofluorescence. The embryos were mounted on glass slides using Mowiol^®^ 4-88 mounting medium. The slides were observed by a LSM880 Zeiss confocal laser microscope with the laser set on λ = 488 nm. Z-stack images of the head and tile scan Z-stack images of whole embryos were taken with a 4 μm interval. Images were then processed using ZEISS ZEN Imaging Software to obtain the maximum intensity projection image. For enhanced green fluorescent protein (eGFP) signal quantification of the diencephalon, 20 to 27 embryos were analyzed three times by drawing the area including the diencephalon. The average axon length of the embryos was assessed by quantifying the length of the 5th-to-16th projection of the spinal cord neurons of 15 embryos for each condition using ImageJ Software’s plugin, NeuronJ [24,25].

### 2.14. Immunohistochemistry and Brightfield Microscopy on Mouse Brain Sections

Newborn pups were dislocated and fix in 4% PFA for 6 h. Two- and 10-month-old mice were anesthetized by intraperitoneal (i.p.) injection of chloral hydrate (400 mg/kg) (Sigma-Aldrich) and were perfused transcardially using a 4% PFA Immunofix solution (05-K01015, Bio-optica, Milan, Italy). After 4 h post-fixation, brains were incubated in a solution of PBS with high salt concentration (NaOH 200 mM, NaH_2_PO_4_ 245 mM, NaCl 0.9%) containing 18% sucrose for at least 24 h, then 30 µm coronal sections were cut with a cryostat (Leica Biosystems, Milan, Italy) and conserved in 60% glycerol. Sections of the *Substantia Nigra* (SN) and striatum were permeabilized with 20% methanol and 5% H_2_O_2_, washed and incubated with primary antibody (TH, AB152, Merck Millipore) overnight (ON) at 4 °C. On the following day, sections were washed and probed with anti-rabbit biotinylated secondary antibody for 45 min at rt. This was followed by gentle washing, incubation with avidin–biotin complex (PK6100, ABC kit, Vector Laboratories, Burlingame, CA, USA) at rt for 45 min and 3,3′-diaminobenzidine (DAB) staining (SK-4100, Vector Laboratories) for 5 min. Finally, sections were washed, dehydrated, mounted with Vectamount mounting medium (H-5000-60, Vector Laboratories) and were observed by means of an inverted light/epifluorescence microscope (Olympus IX50; Olympus, Milan, Italy).

### 2.15. Nigral TH-Positive Stereological Quantification and Analysis of TH Density in the Pars Reticulata (pr) and in Striatal Sections

The number of TH-immunopositive cells was stereologically estimated by double blind cell counting in bright field microscopy using an optical fractionator method, as previously described [16]. Neurons from the SN *pars compacta* (pc) were analyzed with an inverted microscope (Zeiss Axiovert S100 and camera PCO Sensicam) interfaced with a PC running the StagePro module of Image-ProTM Plus software (version 6.2, Media Cybernetics, Inc., Rockville, MD, USA). The entire extension of mesencephalon was cut into sections. Three sections (30 μm thick) were examined every 150 μm in a rostro-caudal extension. TH-immunoreactive cells lateral to the medial terminal nucleus of the accessory optic tract, which defines the medial border of the SN pc were counted. The ventral tegmental area was therefore excluded from these cell counts.

The optical density of TH-positive area of the pr was examined from digitized images using ImageJ software. Brains from five mice (ten sections from each mouse) were analyzed by examining an average of 10 fields per section for 2 and 10 months of age and five sections for newborn pups. Data from nigral TH-positive cell counts and from the analysis of TH-positive area were expressed as % changes versus the mean of wt controls.

The optical density of striatal TH-positive area was examined from digitized images using ImageJ software. Brains from 2 and 10 month-old mice (10 sections from each mouse, *n* = 3 for each group) were analyzed by examining an average of 15 fields per section. Brains from newborn mice (5 sections from each mouse, *n* = 3 for each group) were analyzed by examining an average of two fields per section. Data from striatal TH-positive area were expressed as percent changes versus the mean of the respective wt controls.

### 2.16. Immunocytochemistry

After fixation neurons were subjected to permeabilization with 20% methanol plus endogenous peroxidases inactivation in 5% H_2_O_2_, and then washed and incubated with TH antibody over night at 4 °C. On the following day, cells were washed and probed with anti-rabbit biotinylated secondary antibody for 45 min at rt. This was followed by gentle washing, incubation with avidin–biotin complex at rt for 45 min and DAB staining for 5 min. Finally, sections were washed and observed by means of an inverted light/epifluorescence microscope.

### 2.17. Sholl Analysis on Primary mDN

The Sholl analysis of TH-positive mDN neurons was performed manually using imageJ software. Neurons were randomly chosen and the number of intersections of the neurite tree with increasing circular circles from the center of the soma was counted every 10 µm using the concentric circle mask. The number of branches from the cell body were manually counted and the area was measured by drawing the shape of the soma and averaged.

### 2.18. Morphometric Analyses on TH-Positive iPSCs-Derived mDN

Neurons were fixed using 4% PFA and stained for TH antibody as described above. Digital images of the immunocytochemical assays were captured with an Olympus IX51 microscope connected to an Olympus digital camera and analyzed using ImageJ software. The morphologic indicators of structural plasticity were maximal dendrite length, primary dendrite number and soma area. Three slides per treatment group were examined to obtain measurements from at least 30 neurons.

### 2.19. Clarification of Striatal Tissues and Two-Photon Microscopy

Brains from newborn mice were fixed for 8 h in Immunofix and clarified with the X-Clarity system (Logos Biosystem, South Korea). Briefly, whole brains were polymerized with acrylamide “hydrogel solution” for 3 h at 37 °C with a vacuum followed by the passive clarification for 48 h at 37 °C in “clearing solution”.

Brains were then subjected to deep-tissue TH immunolabeling by staining with TH antibody (dilution 1:100, Merck Millipore) in PBS/0.3% Triton for 3 days, 1 day of washing followed by incubation with anti-rabbit Alexa 488 for 3 days (Jackson Immunoresearch).

Finally, brains were observed by a two-photon upright LSM 880 Zeiss microscope equipped by W Plan-Apochromat 20x/1.0 DIC (UV) VIS-IR M27 75 mm objective. The fluorochrome was excited with the IR laser set at 860 nm. Z-stack were set with the height of the sections scanning = ~1 μm. Images were then reconstructed using Zen lite 2.3 (Carl Zeiss).

### 2.20. Western Blot Analysis

Fresh frozen zebrafish embryos pools (10 embryos for each experimental condition) or tissues from the striatum and midbrain of either Syn III ko or wt mice collected from mouse brains after cervical dislocation were used. Total proteins were extracted with Radioimmunoprepitation Assay (RIPA) buffer made up of 50 mM Tris-HCl pH 7.4, 150 mM NaCl, NP-40 1%, sodium deoxycholate 0.1%, sodium dodecyl sulfate (SDS) 0.1%, 1 mM NaF, 1 mM NaVO4 plus complete protease inhibitor mixture (11873580001, Roche Diagnostics, Mannheim, Germany). Protein concentration in the samples was measured using the Bio-Rad DC^TM^ protein assay kit (5000111, Bio-Rad Laboratories, Milan, Italy). Equal amounts of proteins (30 µg for mouse brain samples, 60 µg for zebrafish embryos samples) were run on 10% polyacrylamide gels and transferred onto polyvinylidene fluoride (PVDF) membrane. Densitometric analysis of the bands were performed using ImageJ Software and all bands were normalized to Glyceraldehyde 3-phosphate dehydrogenase (GAPDH) or α-Tubulin levels as a control of equal loading of samples in the total protein extracts. For densitometry analysis of bands, each experimental condition was performed in quadruplicate and the resulting data were subjected to statistical analysis. The primary antibodies used for WB studies and their working dilutions are listed in Table 1.

### 2.21. Behavioral Tests

#### 2.21.1. Analysis of Spontaneous Head-Tail Coil Spontaneous Movements, Corkscrew Swimming, and Touch-Evoked Tests in Zebrafish Embryos

Zebrafish embryos were injected with *Syn III*-MO, ST-MO (control) and incubated in fish water; non-injected (NI) embryos were used as negative control. Embryos were kept at 28 °C until they reached the correct stage of development to carry out the test. At 21 hpf, 40 injected embryos (ST-MO and *Syn III*-MO, respectively) and NI were observed for head-tail coil spontaneous contractions for 1 min under microscope. The total number of head-tail coil movements (tail flip) of three independent experiments was recorded for each embryo, the average was calculated, and a box diagram was plotted for injected and not injected embryos.

At 72 hpf, 40 embryos for each type were subjected to touch-evoked test, following the protocol [26] with slight modifications. A motility wheel consisting of four concentric circles of increasing diameter (5, 10, 15, 20 mm) was placed under the microscope and centered at the bottom of a 60 mm Ø Petri dish containing fish water. Each embryo was placed in the center of the inner circle and the tail was gently stimulated with a poker tool, the distance it swam in the predetermined concentric circles was recorded. If the embryo could not cross the first circle after multiple attempts (5 attempts), it was determined as incapable of exiting the circle. Once data of the distance swam all 25 embryos of each group were obtained, the percentage of embryos crossing each predetermined concentric circle were calculated.

At 2, 3, and 4 dpf, the corkscrew swimming (spiraling, whirling) movements were analyzed. Briefly, 40 embryos per type were observed for 3 min under the microscope. The total number of corkscrew movements of three independent experiments was recorded for each embryo, the average was calculated, and a box diagram was plotted for injected and NI embryos.

#### 2.21.2. Accelerating Rotarod Test and Pole Test in Adult C57BL/6J and Syn III Ko Mice

The training trials of rotarod test were performed at day 0 by setting a slow speed (5 rpm) on 3 cm diameter 47,650 Rota-Rod (Ugo Basile, Milan, Italy). The animals were placed back and habituated to stay on the drum for 3 min. The learning session is concluded after remaining 180 s on the drum and repeated for at least three times every 30 min since all the trial were completed. The following day the accelerating rotarod protocol was tested by increasing the speed from 4 to 40 rpm up to 300 s and the latency to fall was recorded automatically from the instrument. The number of learning trials, the best trial, and the mean trial, as well as the latency to fall for each trial were reported by examining 4/10 mice per condition.

The pole test was performed by placing the mice head-upward on the top of a vertical pole with a rough surface (2 cm diameter, 50 cm height). The pole was placed in the home cage and a habituation period was waited by placing animals on the cage of the pole 3 min before the test. A learning session was performed for five times every 30 min. The following day, three trials every 30 min were recorded and the time to descend of the best trial and mean trial were evaluated with the maximal severity set at 100 s. Results from 3–7 mice were averaged.

### 2.22. Statistical Analysis

The data are reported as representative of two or more experiments with similar results, depending on the number of embryos analyzed in each experiment. Statistically significant differences between groups were calculated by one-way ANOVA coupled with either Newman-Keuls or Tuckey’s post-comparison tests or Student’s *t*-test. Statistically significance was established at *p* < 0.05.

## 3. Results

### 3.1. Syn III Gene KD Induced an Overt Developmental Delay in Zebrafish Embryos

In zebrafish, *syn3* gene is expressed in midbrain, hindbrain, diencephalon, tegmentum, and dorsal and ventral spinal cord cells during earliest developmental phases [27]. Most of these areas contain dopaminergic neurons [28,29,30,31].

**Figure 1:**
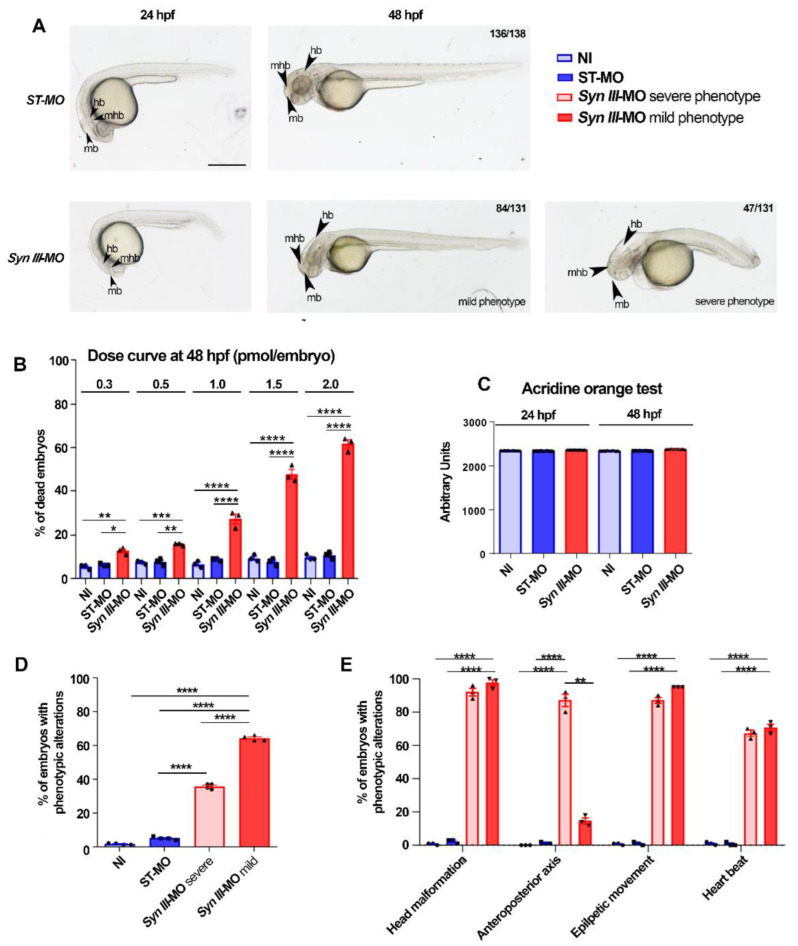
Phenotypic alterations in *Syn III*-MO-injected zebrafish. (**A**) Representative images of ST-MO- or *Syn III*-MO-injected embryos at 24 and 48 hpf. *Syn III*-MO-injected morphants exhibited mild or severe phenotype at 48 hpf. Scale bar, 500 μm. Abbreviations: mb, midbrain; hb, hindbrain; mhb, midbrain hindbrain boundary. (**B**) Ascending doses (in pmol/embryos) of *Syn III*-MO produced significant progressive increase in the percentage of dead embryos that was comparable in ST-MO and control non-injected (NI) embryos. (* *p* < 0.05, ** *p* < 0.01, *** *p* < 0.001, **** *p* < 0.0001 One-way ANOVA + Newman-Keuls, *n* = 3 experimental replicates represent the % mean of dead embryos per group from three experiments including at least 20 embryos per group). (**C**) Acridine orange fluorescence intensity (in arbitrary units) in the embryos injected with 1.0 pmol/embryo of ST-MO or *Syn III*-MO when compared to NI embryos at 24 and 48 hpf (One-way ANOVA + Newman-Keuls. *n* = 4 experimental replicates represent the mean fluorescence intensity per group in four experiments including at least 10 embryos per group). (**D**) At 48 hpf a significantly higher percentage of *syn3* KD morphants exhibited mild the phenotype (**** *p* < 0.0001; one-way ANOVA + Newman-Keuls, *n* = 3 experimental replicates represent the % mean of embryos with phenotypic alterations per group from three experiments including at least 20 embryos per experimental condition). (**E**) At 48 hpf, a significantly higher percentage of mild and severe *Syn III*-MO-injected morphants exhibited head malformations, hyperactive movements, and heartbeat. Anteroposterior axis alterations frequency was significantly increased in the severe phenotype vs. mild phenotype *syn3* KD morphants (**** *p* < 0.0001, ** *p* < 0.05, one-way ANOVA + Newman-Keuls, *n* = 3 experimental replicates represent the % mean of embryos with phenotypic alterations per group from three experiments including at least 20 embryos per experimental condition).

Injection of ascending doses of a splice-inhibiting morpholino to knockdown *syn3* expression (*Syn III*-MO) in zebrafish embryos (1–2 cell stage) induced a significant concentration-dependent increase in mortality at 48 h post-fertilization (hpf) when compared to non-targeting morpholino (ST-MO) injection or NI controls (Figure 1A,B).

The concentration of 1.0 pmol/embryo *Syn III*-MO, inducing morphological changes and about a 25% of embryos death when compared to NI or ST-MO, was selected for subsequent studies as acridine orange analysis also showed that it did not improve cell death at 48 hpf (Figure 1C).

The phenotype induced by *Syn III*-MO (1.0 pmol/embryo) at 48 hpf was scored according to morphological alterations’ percent incidence. The 65% of embryos exhibited a mild phenotype mainly characterized by head malformation and minor anteroposterior axis alterations (smaller eyes and brain with perturbed morphology, shorter and thinner tail, and slightly defective somite development). The remaining 35% of embryos exhibited a severe phenotype (marked alterations of the anteroposterior axis and central nervous system (CNS) structures, cardiac edema, and compromised somite development) (Figure 1A,D). ST-MO-inoculated and NI embryos did not exhibit phenotypic alterations. *syn3* KD affected head morphology, heartbeat and induced hyperactive movements in a similar manner in both phenotypes (Figure 1E).

Mild phenotype larvae were selected as the best model to assess the effect of *syn3* KD.

Gene silencing efficiency in the *Syn III*-MO-injected mild phenotype embryos was assessed by RT-PCR and qrt-PCR. At 48 h and 3 dpf, Syn III was expressed in ST-MO-injected embryos but disappeared in the *Syn III*-MO-injected larvae. In line with the transient effect of morpholino, the *Syn III*-MO gene silencing efficiency was lost at 5 dpf (Figure S1A). These findings were in line with qrt-PCR results showing a significant reduction in Syn III mRNA expression in the *Syn III*-MO-injected larvae, when compared to the ST-MO-injected controls, at 48 hpf (Figure S1B).

**Figure 2:**
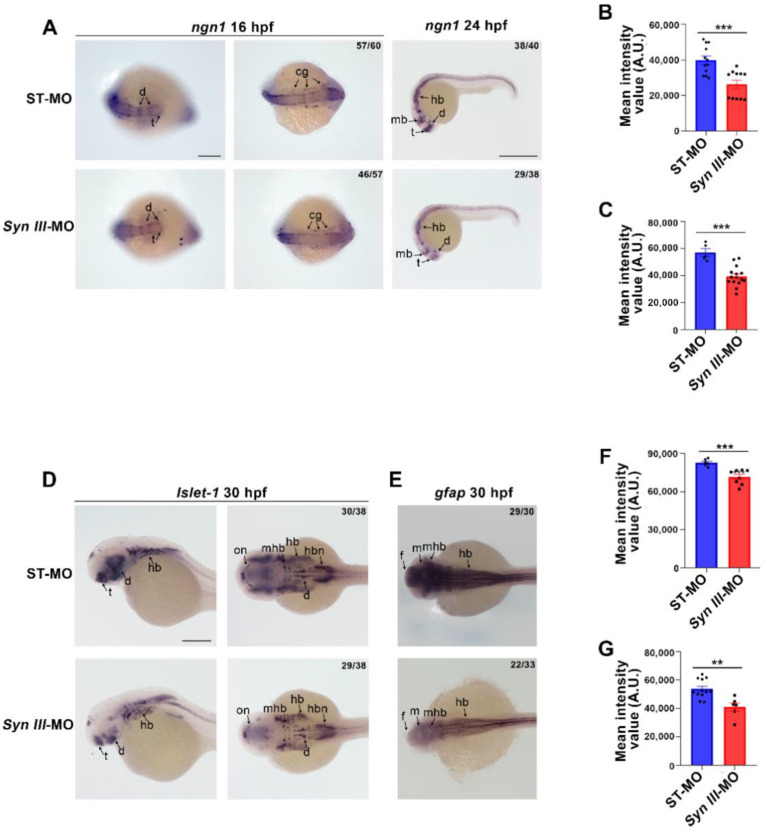
Expression of *ngn1*, *Islet-1* and *gfap* is reduced in *Syn III*-MO injected embryos. (**A**) Representative images of ST-MO and *Syn III*-MO-injected zebrafish embryos hybridized with *ngn1* at 16 hpf and 24 hpf. Abbreviations: mb: midbrain, hb, hindbrain; cg, cranial ganglia; t, telencephalon; d, diencephalon. 16 hpf scale bar, 200 μm; 24 hpf scale bar, 500 μm. (**B**,**C**) Quantification of *ngn1* signal at 16 hpf (**B**) and 24 hpf (**C**) as mean intensity values in arbitrary units (A.U.). *Syn III*-MO-injected embryos showed a significant decrease in the mean intensity value when compared to control embryos (*** *p* < 0.001, unpaired Student’s *t*-test, *n* = 12 embryos per group). (**D**,**E**) Representative images showing 30 hpf embryos hybridized with *Islet-1* (**D**) and *gfap* (**E**), scale bar = 200 μm for all the panels. Abbreviations: d, diencephalon; f: forebrain; hb, hindbrain; hbn, hindbrain neurons; mhb, midbrain hindbrain boundary; mb, midbrain; on, optic nerve; t, telencephalon. (**F**,**G**) Quantification of *Islet-1* (**F**) and *gfap* (**G**) signal expressed as mean intensity values. *Syn III*-MO-injected embryos exhibited a significant decrease of both *Islet-1* ((**F**), *** *p* < 0.001 unpaired Student’s *t*-test) and *gfap* when compared to control embryos ((**G**), ** *p* < 0.01, unpaired Student’s *t*-test). *n* = 6–15 embryos per group).

Then, we used WISH to study the expression of *ngn1*, which is expressed by basal forebrain dopaminergic neuronal progenitors either in the pro-neural clusters detectable at 16 hpf or in the ventricular proliferative zones and mitotic cells at 24 hpf [32,33]. At 16 hpf, *Syn III*-MO-injected morphants exhibited a significant reduction in the expression of *ngn1* in main anterior developing structures, such as cranial ganglia and diencephalic neurons (Figure 2A,B). At 24 hpf, the decrease of *ngn1* expression associated with *syn3* KD involved basal forebrain (telencephalon, diencephalon, midbrain, and hindbrain) (Figure 2A,C).

WISH-based analysis of LIM/homeobox gene *Islet-1*, playing a relevant developmental and regulatory function in dopaminergic neurons [34], showed a significant reduction in the expression of this gene in diencephalon, telencephalon and hindbrain of *Syn III*-MO-injected embryos, when compared to ST-MO controls, at 30 hpf (Figure 2D,F).

Since synapsins are also expressed within developing astrocytes [35] and astrocyte-derived synapsin I (Syn I) can promote neurite outgrowth [36], we also checked whether *syn3* KD could impact on *gfap* expression at 30 hpf, when *gfap* is expressed in neuronal and glial precursor cells, although it can be considered a specific glial marker from around 72 hpf onward [37]. *Syn III*-MO-injected embryos exhibited a significant reduction in *gfap* expression when compared to ST-MO-injected controls (Figure 2E,G).

To further characterize the involvement of Syn III in zebrafish neurogenesis and neurodevelopment, we then used two transgenic lines expressing eGFP, under the control of neuronal differentiation 1 (*neurod1*), referred as Tg(*neurod1*:eGFP) [38], and *ngn1* which is a *neurod1* upstream regulator, referred as Tg(*ngn1*:eGFP) [39]. These promoters are involved in neuronal development and their expression correlates and partially overlaps in many zebrafish brain regions, with *ngn1* being more restricted toward the ventricular proliferative zones and *neurod1* extending more laterally [40].

**Figure 3:**
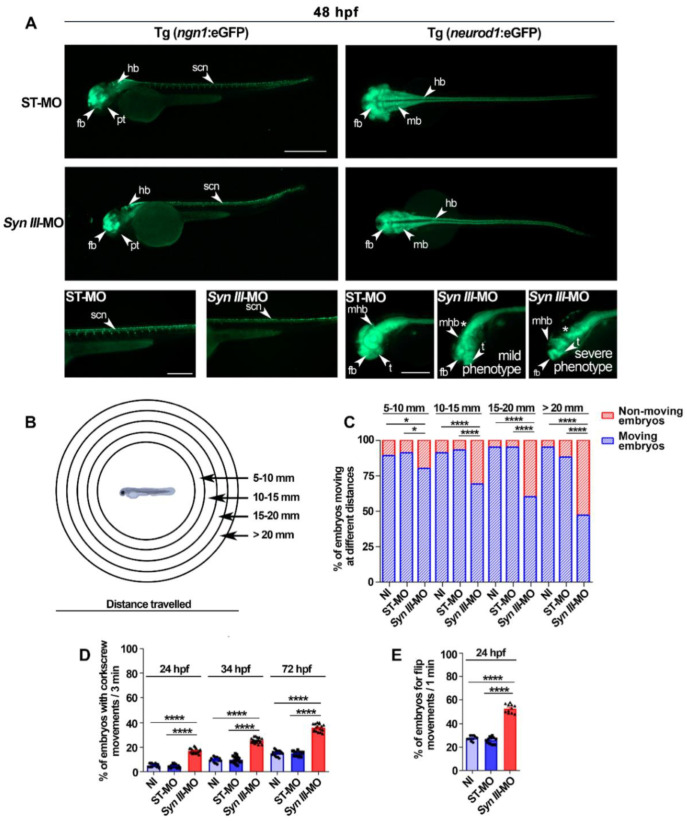
*Syn III*-MO injection affects Tg(*ngn1*:eGFP) and Tg(*neurod1*:eGFP) embryos neurodevelopment and behavior. (**A**) Representative lateral (left panels) and dorsal (right panels) views of *ngn1* and neurod1 dependent eGFP fluorescence in the 48 hpf ST-MO- or *Syn III*-MO-injected Tg(*ngn1*:eGFP) and Tg(*neurod1*:eGFP) embryos, respectively. Full lateral/dorsal view scale bar, 500 μm. Tail/head magnifications scale bars = 250 μm. Asterisk indicates the midbrain/hindbrain regions exhibiting a clear reduction of the eGFP signal in the morphants. Abbreviations: fb, forebrain; hb, hindbrain, mb: midbrain; mhb, midbrain hindbrain boundary; t, telencephalon; pt, posterior tuberculum; scn, spinal cord neurons. (**B**) Schematic representation of the touch-evoked test. (**C**) Swimming performance of embryos at 72 hpf in the touch-evoked test. A significant progressive decrease in the % of *syn3* KD morphants when compared to NI or ST-MO-injected embryos was detected (* *p* < 0.05, **** *p* < 0.0001, one-way ANOVA + Newman-Keuls, results are representative of three independent experiments with 40 embryos per group). (**D**) A significant increase in the percent of *Syn III*-MO-injected embryos exhibited corkscrew movement when compared to NI or ST-MO-injected embryos was observed at 24, 48 or 72 hpf (**** *p* < 0.0001, one-way ANOVA + Newman-Keuls, results are representative of three independent experiments with ≅ 10 embryos per group). (**E**) A significant increase in flipping movement was observed in the *Syn III*-MO-injected embryos when compared to NI or ST-MO-injected ones at 24 hpf (**** *p* < 0.0001, one-way ANOVA + Newman-Keuls, results are representative of three independent experiments with 10 embryos per group).

In ST-MO-injected Tg(*ngn1*:eGFP) control embryos at 48 hpf, the eGFP signal localized in ventral and dorsal diencephalon, posterior tuberculum, and trunk motor neurons [39]. In *Syn III*-MO-injected Tg(*ngn1*:eGFP) embryos the eGFP signal was not detectable in spinal cord motor neurons projections, supporting a neurodevelopmental deficit (Figure 3A). Analysis of eGFP localization in the ST-MO-injected and NI Tg(*neurod1*:eGFP) control embryos confirmed the localization in tegmentum, diencephalon, hindbrain, and lateral line ganglia (Figure 3A and Figure S1C), as previously reported [38]. Tg(*neurod1*:eGFP) exposed to *syn3* KD showed a decreased eGFP signal in telencephalic and diencephalic regions where the dopaminergic neurons localize (Figure 3A). The decrease of eGFP-positive signal in the head of the *Syn III*-MO- vs. ST-MO-injected Tg(*neurod1*:eGFP) embryos was corroborated by light-sheet microscopy-based 3D visualization (Videos S1 and S2).

Since brain and motor neurons development decreased in the embryos exposed to *syn3* KD and brain dopaminergic projections promote motor neuron generation in the developing zebrafish [41], we also performed time-lapse-based light-sheet microscopy acquisitions of whole *Syn III*-MO vs. ST-MO-injected Tg(*neurod1*:eGFP) larvae from 19 to 24 hpf. These confirmed that the neurodevelopmental decrease observed in the embryos exposed to *syn3* KD involved both head and trunk neurons (Videos S3 and S4).

Finally, we evaluated whether *syn3* KD-induced reduction of neuronal development could affect zebrafish motility by studying touch-evoked swimming at 3 dpf. The percentage of *Syn III*-MO-injected Tg(*neurod1*:eGFP) embryos exhibiting reduced swimming distance progressively increased within the interval between 5 and 10 mm and >20 mm (Figure 3B,C).

By analyzing corkscrew swimming, a spiral swimming with increased speed in uncoordinated direction, at 2, 3 and 4 dpf we found a significant increase in the *Syn III*-MO-injected Tg(*neurod1*:eGFP) embryos with respect to ST-MO-injected and NI controls (Figure 3D). The percentage of *Syn III*-MO-injected embryos with flip movements at 24 hpf was also significantly increased (Figure 3E).

### 3.2. Rat Syn III mRNA Expression Rescued the Neurodevelopmental Decrease in Syn III-MO-Injected Zebrafish Embryos

By sequence homology analysis of zebrafish *syn3* and its human, rat, and mouse orthologs (Figure S2A), we found that the percent identity in amino acid sequence between *Danio Rerio* and either *Mus Musculus* or *Rattus Norvegicus* was 66%, while it raised to 67% with *Homo Sapiens*. Sequence homology reached 92% between rat or mouse and human and 98% between rat and mouse (Figure S2B). A highly conserved synteny between the region surrounding human SYN3 on chromosome 22, mouse chromosome 10 and the region surrounding *Danio Rerio* homologue on chromosomes 4 was detected (green boxes, Figure S2C). This supports a positive evolutionary pressure on the Syn III gene as previously reported [27].

**Figure 4:**
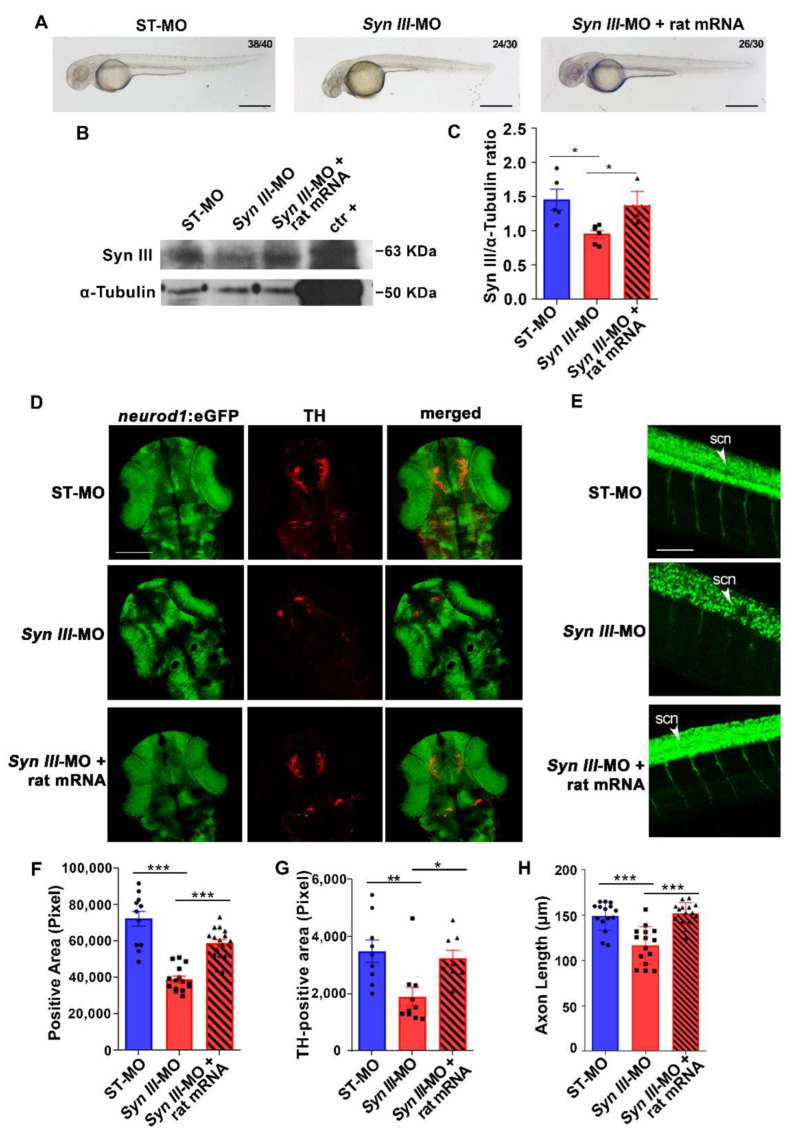
The neurodevelopmental deficits rescued by rat Syn III mRNA expression of *Syn III*-MO-injected Tg(*neurod1*:eGFP) embryos involve TH-positive dopaminergic neurons. (**A**) Representative images showing embryos injected with non-silencing ST-MO, *Syn III*-MO or *Syn III*-MO and rat mRNA (*Syn III*-MO + rat mRNA). Scale bars, 100 μm. (**B**) Representative WB from protein extracts of ST-MO-, *Syn III*-MO- or *Syn III*-MO + rat mRNA-injected Tg(*neurod1*:eGFP) embryos. The positive control is a protein extract obtained from the striatum of twelve-month-old C57BL/6J wt mice. Alpha-Tubulin bands are representative of the equal loading of samples. (**C**) Graphs show the ratio between the optical density (o.d.) of Syn III and α-Tubulin of ST-MO, *Syn III*-MO, and *Syn III*-MO + rat mRNA-injected Tg(*neurod1*:eGFP) embryos. Syn III was significantly reduced in the embryos exposed to *syn3* KD when compared to either control or *Syn III*-MO + rat mRNA (* *p* < 0.05, One-way Anova + Newman-Keuls, *n* = 5 zebrafish protein pools per experimental condition). (**D**) Representative images showing TH immunostaining on 48 hpf Tg(*neurod1*:eGFP) embryos injected with ST-MO, Syn III-MO, or *Syn III*-MO + rat mRNA. Scale bar, 200 μm. (**E**) Representative images showing lateral spinal cord views of 48 hpf Tg(*neurod1*:eGFP) embryos injected with ST-MO, *Syn III*-MO, or *Syn III*-MO + rat mRNA. Scale bar, 200 μm. Abbreviations: scn, spinal cord neurons. (**F**) Quantification of the eGFP-positive area in the diencephalon. *** *p* < 0.001, one-way ANOVA + Newman-Keuls. *n* = 11–15 zebrafish embryos per group. (**G**) Graph shows the TH-immunopositive area (in pixel) of the 48 hpf Tg(*neurod1*:eGFP) embryos injected with ST-MO, *Syn III*-MO, or *Syn III*-MO + rat mRNA. Rat Syn III expression rescued the developmental deficits of TH-positive neurons in *syn3* KD embryos (** *p* < 0.01, * *p* < 0.05; One-way ANOVA + Newman-Keuls. *n* = 8–10 zebrafish embryos per experimental condition). (**H**) Graph shows the spinal cord neuron axon length (in µm) of ST-MO-, *Syn III*-MO- or *Syn III*-MO + rat mRNA-injected Tg(*neurod1*:eGFP) embryos at 48 hpf. Injection of rat Syn III rescued the reduction in spinal neurons axonal length in *syn3* KD embryos (*** *p* < 0.001 *Syn III*-MO or *Syn III*-MO + rat mRNA vs. control. One-way ANOVA + Newman-Keuls. *n* = 15 zebrafish per group).

To corroborate the specificity of *syn3* KD on neuronal development and assess whether Syn III neurodevelopmental function is conserved in vertebrates, we thus probed whether the expression of rat Syn III mRNA (produced by injecting 100 pg/embryo of pCS2+ plasmid transducing rat Syn III mRNA) could rescue the abnormal neuronal phenotype in the *Syn III*-MO-injected Tg(*neurod1*:eGFP) embryos. In particular, we used rat Syn III mRNA for rescue experiments to assess whether the protein sequences impinging on the neurodevelopmental function were conserved in rodents and zebrafish. Rat Syn III mRNA expression redeemed the morphological changes in the phenotype of *Syn III*-MO-injected Tg(*neurod1*:eGFP) embryos (Figure 4A) and rescued the significant reduction in zebrafish Syn III protein levels observed in the *Syn III*-MO Tg(*neurod1*:EGFP)-injected embryos at 48 hpf (Figure 4B,C). Rat Syn III mRNA expression also rescued the reduction in brain development observed in the *Syn III*-MO injected Tg(*neurod1*:eGFP) embryos at 48 hpf (Figure 4D,F).

We also found that rat Syn III-mRNA expression in the Tg(*neurod1*:eGFP) larvae at 48 hpf rescued the significant reduction in the TH-positive area observed in the ventral telencephalon and dorsal diencephalon of *Syn III*-MO-injected Tg(*neurod1*:eGFP) embryos (Figure 4D,G), supporting a recovery of dopaminergic neurons development deficits.

Since light-sheet microscopy-based time-lapse imaging suggested that *Syn III*-MO-injected Tg(*neurod1*:eGFP) embryos also exhibited a reduced spinal neuron development, we assessed axonal length in the spinal cord. We found a significant decrease in the length of the axons innervating the 5th–16th somite in the Tg(*neurod1*:eGFP) embryos injected with *Syn III*-MO, when compared to ST-MO-injected controls (Figure 4E,H). This was rescued by rat Syn III mRNA expression (Figure 4E,H).

### 3.3. Syn III Affects the Development of Mouse SN Neurons In Vivo

In mammals, Syn III controls early maturation of short projecting cortical and hippocampal neurons [6,15,42,43,44], but we ignore whether it also affects the development of mDN, which are characterized by long-distance and extensively arborized multi-synaptic axonal projections [45,46,47,48].

We thus evaluated whether newborn, 2- or 10-month-old Syn III ko mice exhibited nigrostriatal mDN developmental impairment. Western blot analysis confirmed absence of Syn III expression in the SN and striatum of Syn III ko mice (Figure S3). Interestingly, this also demonstrated an age-dependent decrease in Syn III levels in the wt mice (Figure S3A–D). As Synapsins act in heterodimeric complexes and exhibit an elevated sequence similarity [6,49], we also checked whether the deficiency of Syn III could induce homeostatic compensatory changes in either Syn I or Synapsin II (Syn II) expression (Figure S3E,F). Though Syn II levels were comparable in wt and Syn III ko mice, Syn I levels resulted significantly reduced in the midbrain and striatum of Syn III ko mice at 10 months of age (Figure S3G,H), hinting that the absence of Syn III can affect Syn I expression in the long term.

**Figure 5:**
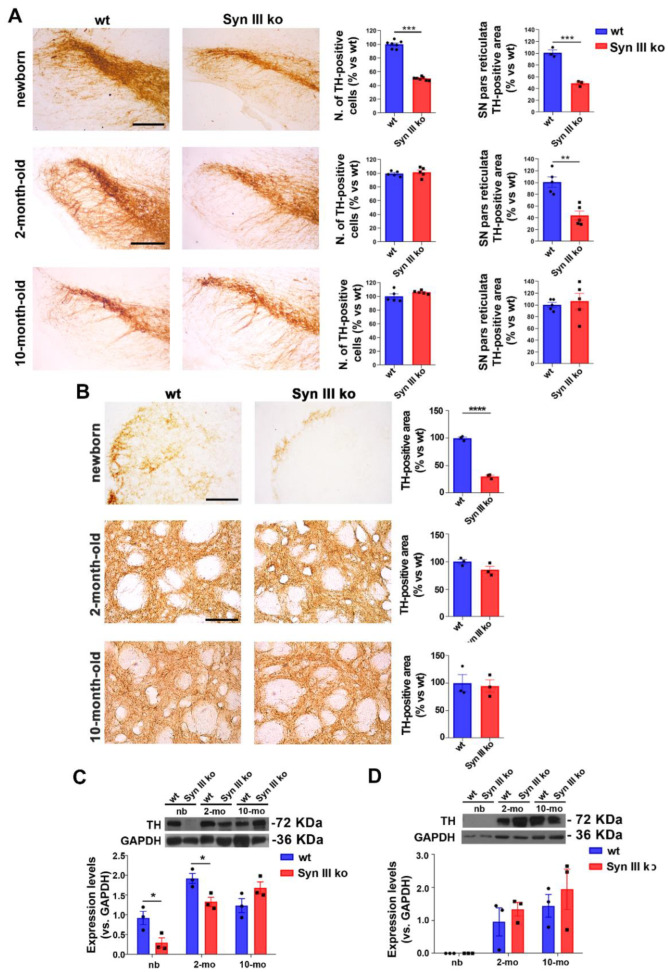
Reduction of nigrostriatal neurons development in newborn Syn III ko mice is progressively recovered in adult mice. (**A**) Graphs show the percent changes in SN TH-positive cell numbers of Syn III ko vs. wt mice evaluated by stereology at 0, 2 and 10 months of age. Newborn mice exhibited a significant reduction of TH-positive neurons (*** *p* < 0.001, unpaired Student’s *t*-test, *n* = 7 per group). The analysis of TH-immunopositive area in the pr of wt and Syn III ko mice (as percent change vs. age-matched wt mice) showed that the absence of Syn III reduced this parameter in both newborn and 2-month-old mice (*** *p* < 0.001, ** *p* < 0.01, unpaired Student’s *t*-test, *n* = 3–5 animals per group). Scale bar newborn mice: 100 μm; scale bar 2 and 10 month-old mice: 150 μm. (**B**) Representative images showing striatal TH-positive fibers of newborn, 2-month- or 10-month-old wt and Syn III ko animals. Graphs show the percent changes in the striatal TH-positive area in the Syn III ko mice vs. wt mice (**** *p* < 0.0001, unpaired Student’s *t*-test, *n* = 3 animals per group). Scale bar newborn mice: 120 μm; scale bar 2 and 10 month-old mice: 200 μm. (**C**) Representative images showing TH-immunopositive bands from WB analysis of Syn III ko or wt mouse midbrain protein extracts. Graph shows that TH levels were significantly reduced in the midbrain of newborn (nb) and 2-month-old Syn III ko mice when compared to wt littermates. No difference in TH levels was observed at 10 months of age. Data expressed as TH protein levels normalized vs. GAPDH expression (* *p* < 0.05, two-way ANOVA + Bonferroni’s post-test, *n* = 3 animals per group). (**D**) WB of striatal protein extracts from Syn III ko and wt mice showed that TH protein levels did not exhibit significant changes at 0 (nb), 2 and 10 months of age. Two-way ANOVA + Bonferroni’s post-test (*n* = 3 animals per group).

We then assessed the percentage changes of the number of TH-positive cells in the SN and their pr dendritic arborizations in newborn, 2 and 10 month-old wt and Syn III ko mice. Newborn Syn III ko mice exhibited a significant 50% and 52% reduction in the number of SN TH-positive cells and pr TH-positive processes vs. wt mice, respectively (Figure 5A). By 3D reconstruction of TH-positive cells in two-photon-based acquisition of whole SN from newborn mouse brains subjected to X-clarity-based clearing and deep-tissue fluorescence immunolabeling, we confirmed the reduction in mDN development in the SN of newborn Syn III ko (Video S5) when compared to wt mice (Video S6).

While the reduction in SN neurons was recovered at 2 and 10 months of age, the decrease of pr TH-positive processes was still present at 2 months of age, but disappeared in older mice (Figure 5A). The recovery of the proper number of dopaminergic SN neurons at 2 months and of striatal processes in older mice could be likely ascribed to the fact that the neurodevelopmental function of Syn III is confined in a very early time window, in line with the very early peak of Syn III expression during neuronal development [6,15,44]. In later developmental stages, Syn III does not play a major role, while Syn I and Syn II affect synapse elongation and maturation, axonal branching, neurite elongation and outgrowth [6].

The decrease of SN dopaminergic neurons in Syn III ko newborn mice was reflected by a 70% decrease in striatal TH-positive projections that was recovered from 2 months of age (Figure 5B).

These findings collimated with the WB-based evaluation of TH levels in the midbrain (Figure 5C) and striatum (Figure 5D).

Finally, we assessed the motor abilities of Syn III ko mice (Figure S4A,B). To probe the acquisition of skilled behavior minimizing the learning feature in the rotarod and pole tests, wt and Syn III ko mice at 2 or 10 months of age were employed in training trials on the first day and both the best trial and mean trial of the second day were evaluated. No differences between the basal motor activity of wt and Syn III ko animals was detected at 2 or 10 months of age (Figure S4A,B).

### 3.4. Syn III Deletion Hinders Early Mouse Primary mDN Development

**Figure 6:**
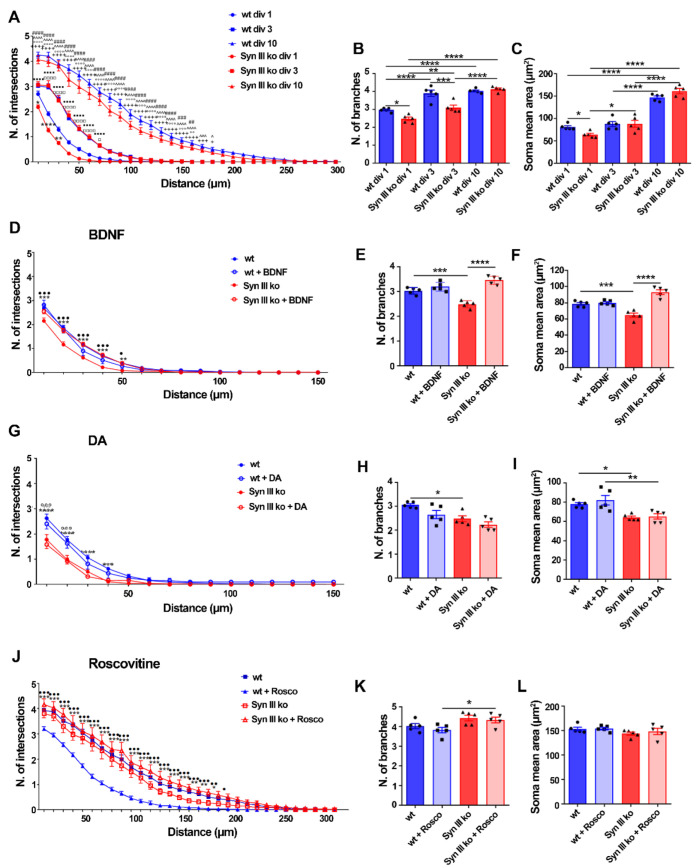
Syn III ko TH-positive mesencephalic neurons exhibit neurodevelopmental deficits involving BDNF and Cdk5 signaling. (**A**,**D**,**G**,**J**) Sholl analysis assessing the number of intersections. (**A**) A significant decrease in the number of intersections between the neuronal projections of primary midbrain TH-positive cells and concentric circles in div 1 Syn III ko neurons when compared to wt neurons (**** *p* < 0.0001, ** *p* < 0.01, * *p* < 0.05). A significant increase in the number of intersections was observed in div 10 when compared to div 3 (^ *p* < 0.05, ^^^ *p* < 0.001, ^^^^ *p* < 0.0001) or div 1 wt neurons (+ *p* < 0.05, +++ *p* < 0.001, ++++ *p* < 0.0001) as well as in div 10 to when compared to div 3 (## *p* < 0.01, ### *p* < 0.001, #### *p* < 0.0001) or div 1 Syn III ko neurons (°° *p* < 0.01, °°° *p* < 0.001, °°°° *p* < 0.0001). In Syn III ko neurons a significant increase in the number of intersections at div 3 when compared to div 1 neurons was also detected (■■■■ *p* < 0.0001, □ *p* < 0.05, □□□□ *p* < 0.0001, in wt neurons). (**D**) Sholl analysis showed that BDNF treatment restored the reduction in the number of intersections in Syn III ko but not in the wt TH-positive cells (*** *p* < 0.001, ** *p* < 0.01, Syn III ko vs. wt untreated neurons and ••• *p* < 0.001, • *p* < 0.05, untreated vs. treated Syn III ko neurons. (**G**) DA treatment was not effective in improving the number of intersections in Syn III ko TH-positive cells (*** *p* < 0.001 and **** *p* < 0.0001 vs. wt untreated neurons and °°° *p* < 0.001, vs. wt neurons. (**J**) Roscovitine treatment reduced number of intersections of wt TH-positive cells, but not that of Syn III ko neurons (*** *p* < 0.001, ** *p* < 0.01, * *p* < 0.05, untreated vs. treated wt neurons and ••• *p* < 0.001, •• *p* < 0.01, • *p* < 0.05, wt vs. Syn III ko neurons. Two-way ANOVA + Bonferroni’s post-test). *n* = 5 means that plotted values are the mean values deriving from five experiments analyzing 30 TH-positive cells from primary midbrain neuronal cells cultures of either Syn III ko or wt mice. (**B**,**C**) TH-positive-neurons from Syn III ko showed a significant reduction in the number of branches stemming from the primary midbrain neuronal cell bodies at div1 (* *p* < 0.05) and div 3 (*** *p* < 0.001) when compared to wt mice. A significant decrease of the mean soma area of TH-positive primary midbrain neurons in div 1 Syn III ko when compared to wt cells was detected (* *p* < 0.05). One-way ANOVA + Newman-Keuls multiple comparison test). *n* = 5 means that plotted values are the mean values deriving from five experiments analyzing 30 TH-positive cells from primary midbrain neuronal cultures of Syn III ko or wt mice (**** *p* < 0.0001, ** *p* < 0.01). (**E**,**F**) BDNF treatment also recovered the reduction in the number of branches and mean soma area of Syn III ko neurons (*** *p* < 0.001, Syn III ko vs. wt untreated neurons, **** *p* < 0.0001, untreated vs. treated Syn III ko neurons. One-way ANOVA + Newman-Keuls post-test *n* = 5 means that plotted values are the mean values deriving from five experiments analyzing 30 TH-positive cells from primary midbrain neuronal cells cultures of either Syn III ko or wt mice. (**H**,**I**) DA treatment did not either improve the branching or mean soma are of the TH-positive midbrain neurons from Syn III ko mice (* *p* < 0.05, Syn III ko vs. wt untreated neurons, ** *p* < 0.01, Syn III ko vs. wt DA-treated neurons, one-way ANOVA + Newman-Keuls). (**K**,**L**) The treatment of div 3 primary mouse midbrain neurons with Roscovitine for 24 h did not affect the number of branches and the mean soma area of wt and Syn III ko neurons (* *p* < 0.05). One-way ANOVA + Newman-Keuls multiple comparison test. *n* = 5 means that plotted values are the mean values deriving from five experiments analyzing 30 TH-positive cells from primary midbrain neuronal cultures of Syn III ko or wt mice.

Midbrain neurons were dissected from 13 days wt and Syn III ko embryos and cultured for 1, 3 or 10 div as previously described [2]. Dendritic branching of TH-positive neurons was evaluated using Sholl analysis [50], showing a significant decrease in the number of intersections between neuronal dendritic projections and concentric circles in Syn III ko when compared with wt neurons at div 1 (Figure 6A and Figure S5B,C). These differences were abated at 3 and 10 div (Figure 6A). Syn III ko neurons also exhibited a significant decrease in branches numbers at div 1 and 3 (Figure 6B), and in the soma area at div 1 (Figure 6C).

As BDNF is required for the establishment of the proper number of dopaminergic neurons in the SN [51], which were reduced in the Syn III ko newborn mice, we evaluated whether the observed early deficits in mDN development at div 1 can be restored by 24 h BDNF (10 ng/mL) treatment. BDNF-treated Syn III ko TH-positive primary midbrain neurons exhibited several intersections, branches, and a mean soma area comparable to those of wt cells (Figure 6D–F and Figure S5D). BDNF treatment did not influence these parameters in wt TH-positive neurons (Figure 6D–F), suggesting that the Syn III ko mDN neurons exhibit BDNF receptors hypersensitivity, a phenomenon which could result from a lower BDNF production [52]. In agreement, we found a significant reduction of both prepro-BDNF and mature BDNF levels in the midbrain of newborn Syn III ko mice vs. wt littermates (Figure S5A).

The number of intersections, branches or mean soma area of either Syn III ko or wt mouse TH-positive primary mDN was not influenced by 0.1 µM DA treatment (Figure 6G–L and Figure S5E), in line with evidence supporting that mDN do not express mature and functional DA autoreceptors at div 1 [53,54].

Syn III-mediated promotion of primary cortical neurons maturation at div 3 are improved upon its phosphorylation by Cdk5 [15], which is also involved in BDNF-induced dendritic growth [55]. We thus evaluated whether the neuronal arborization of Syn III ko or wt primary mDN was differentially affected by the treatment with the Cdk5 inhibitor roscovitine. To probe the involvement of early Cdk5 inhibition on neuronal maturation, wt or Syn III ko mDN at div 3 were treated with 1 µM roscovitine for 24 h and subjected to Sholl, branching and soma size analysis at div 10. Roscovitine significantly reduced the number of intersections without affecting the number of branches or mean soma area of primary TH-positive mDN from wt mice, but had no effect on Syn III ko neurons (Figure 6J–L and Figure S5F), supporting that Cdk5-dependent control of mDN dendritic arborization is disrupted in the absence of Syn III.

### 3.5. Early Maturation of Human iPSCs-Derived DA Neurons Is Altered by Syn III Gene Silencing

**Figure 7:**
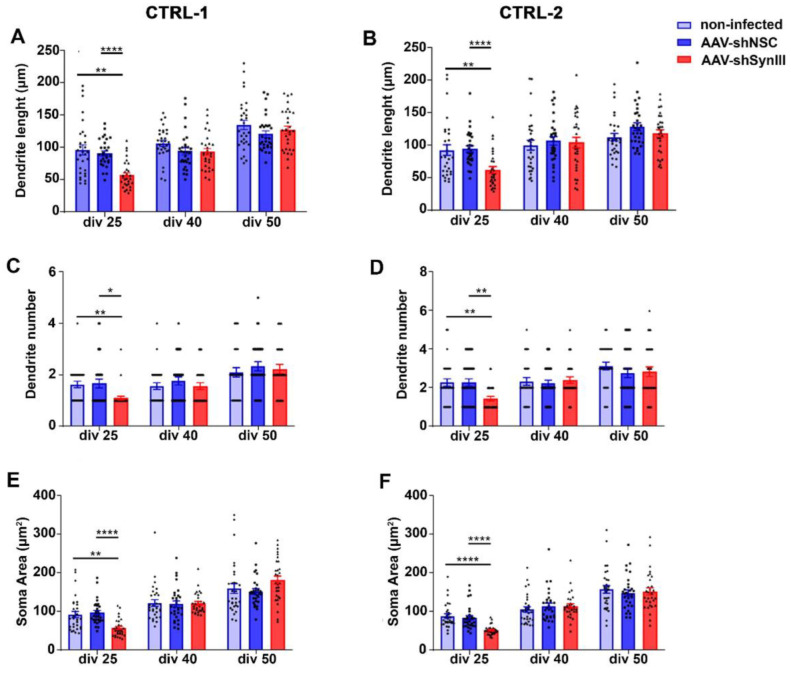
AAV-mediated Syn III RNAi impaired early iPSCs-derived mDN development. (**A**,**B**) Graphs show primary dendrite length analysis (expressed in µm) in control non-infected AAV-shSynIII- or AAV-shNSC-infected mDN produced from CTRL-1 (**A**) and CTRL-2 (**B**) iPSCs clones at div 25, 40 and 50. At 25 div AAV-shSynIII-infected mDN showed a statistically significant decrease in primary dendrite length when compared to NI and AAV-shNSC-infected cells (** *p* < 0.01, **** *p* < 0.001; two-way ANOVA + Bonferroni’s post-test). No changes were observed at div 40 or 50. *n* = 30 cells per each experimental condition. (**C**,**D**) Graphs show dendrites number in control and AAV-shSynIII- or AAV-shNSC-infected mDN produced from CTRL-1 (**C**) and CTRL-2 (**D**) iPSCs clones at div 25, 40 and 50. A significant decrease in the number of dendrites at div 25 was observed in the AAV-shSynIII-infected mDN when compared to both control and AAV-shNSC-infected cells (* *p* < 0.05, ** *p* < 0.01; two-way ANOVA + Bonferroni’s post-test). No changes were observed at div 40 or 50. *n* = 30 cells per each condition. (**E**,**F**) Graphs show the analysis of soma area of mDN (expressed in µm2) from control and AAV-shSynIII- or AAV-shNSC-infected mDN produced from CTRL-1 (**E**) and CTRL-2 (**F**) iPSCs clones at div 25, 40 and 50. A significant decrease in the mean soma area was detected in the AAV-shSynIII-infected mDN at div 25 when compared to control (** *p* < 0.01 and **** *p* < 0.0001) or AAV-shNSC-infected cells (**** *p* < 0.0001). Two-way ANOVA + Bonferroni’s post-test). No changes were observed at div 40 or 50. *n* = 30 cells per each condition.

Finally, we evaluated the involvement of Syn III in the early developmental phases of human iPSC-derived dopaminergic neurons from two healthy subjects [17,18], referred to as CTRL-1 and CTRL-2, exposed to Syn III gene silencing. iPSCs were infected with an AAV expressing either shRNA silencing SYN3 (AAV-shSynIII) or non-silencing RNA sequences (AAV-shNSC) and differentiated until day 50 into a neuronal population enriched in DA neurons as previously described [56,57]. The average maximal length of primary dendrite (from the soma to the first ramification), number of dendrites and soma area of TH-positive neurons were analyzed in each line by computer-assisted morphometry [18] at three different time points (div 25, div 40, div 50) (Figure 7A–F and Figure S6A,B). Syn III gene silencing was maintained throughout the differentiation process, as confirmed by WB analysis (Figure S6C–F). At div 25, TH-positive neurons from AAV-shSynIII exhibited reduced primary dendrite length, dendritic arborization and decreased soma area as compared to both AAV-shNSC and non-infected TH-positive neurons. At day 40 and day 50 the morphology of TH-positive neurons from AAV-shSynIII were comparable to that of both AAV-shNSC and non-infected TH-positive neurons (Figure 7).

## 4. Discussion

Our results support that Syn III controls the very early stages of dopaminergic neuron development and that this function is preserved in vertebrates, in line with the very early and transient increase in Syn III expression during neuronal development [6].

In zebrafish, *syn3* KD induced an overall reduction in neuronal development as supported by the decreased expression of *ngn1*, *Islet-1* and *gfap*. Interestingly, ADHD causal genes are highly expressed in both fetal astrocytes and neurons [58]. Astrocyte alterations can underlie ADHD manifestations, and proper astrocyte activity is relevant for dopaminergic neurons function [59,60,61,62]. Therefore, the here reported reduction in *gfap* expression in common neuronal/astrocyte precursors of *syn3* KD zebrafish embryos, hints that human SYN3 polymorphisms may also lead to a decrease in astrocytes development, potentially contributing to alterations in DA-evoked synaptic regulation and ADHD onset.

The *syn3* KD larvae exhibited a marked decrease in TH-positive neurons and ADHD-like motor alterations similar to that developed by zebrafish embryos subjected to the chemogenetic ablation of dopaminergic neurons [63], with a progressive reduction of overall motility but increased uncoordinated movements. The dopaminergic developmental deficits of *syn3* KD zebrafish embryos were paralleled by a reduction in spinal cord motor neurons development. Although we cannot exclude that this can be directly ascribed to Syn III gene KD, the fact that spinal cord motor neurons development is stimulated by brain DA [41] suggests that the reduced dopaminergic neurons development could also be at the basis of this phenomenon. In line with the elevated sequence homology observed between zebrafish, mouse, rat, and human Syn III-encoding ortholog genes, the developmental impairment associated with *syn3* KD was rescued by the expression of rat Syn III mRNA. In particular, the use of rat Syn III mRNA for rescue experiments allowed to disclose that the Syn III sequences that are relevant for the dopaminergic neurodevelopmental function of the protein were conserved in zebrafish and rodents.

Consistently, by studying mouse Syn III ko TH-positive mDN, we observed that Syn III deficiency reduces their very early development (in 1–3 div embryonic neuronal cultures and newborn animals). The decrease of TH-positive SN neurons number and striatal fibers density is recovered in two month-old Syn III ko mice, though they still exhibit a decrease in the dopaminergic pr projections, which is later retrieved at 10 months of age.

The decrease of SN pr projections at two months of age was not associated with motility deficits, in line with findings describing that Syn III ko mice do not exhibit overt sensory and motor functions alterations [42]. However, Syn III ko mice manifest a behavioral phenotype that is highly reminiscent of ADHD, with social transmission of food preference and object recognition deficits in the absence of anxiety or depressive-like behaviors [42]. Interestingly, we previously demonstrated that Syn III ko mice do not present motility improvement following acute i.p. administration of MPH [11,12], in agreement with our findings supporting that MPH can bind Syn III and with evidence showing that the efficacy of MPH is perturbed in ADHD patients with Syn III polymorphisms [10,12]. Still, consistently with the reduction of cocaine-induced facilitation of DA release in SN pr brain slices from Syn III-deficient mice [1], we also found that these animals do not even present a locomotor response following acute cocaine administration [11]. The impaired response to dopaminergic stimulant drugs of Syn III ko mice may very well reflect an abnormal synaptic function/basal ganglia organization deriving from the mDN developmental delay. Indeed, Syn III ko mice exhibit enhanced electrical stimulation-evoked DA release in the striatum in the absence of morphological alterations in the density or distribution of synaptic vesicles in neurons, supporting that Syn III acts as a negative regulator of phasic dopaminergic neurotransmission without altering synaptic vesicles organization at striatal dopaminergic terminals [1,16].

The activity of SN dopaminergic neurons is physiologically inhibited by pr γ-aminobutirric acid (GABA) afferents [64,65,66], which can finely control the timing of phasic DA signals in the striatum [67]. Mice deficient in the Tal1 gene, encoding a transcription factor that promotes GABAergic differentiation in midbrain dopaminergic nuclei, display ADHD-like behavioral features [68]. Therefore, the delay of pr dendritic development observed in Syn III ko mice may produce deficits in the establishment of input connections to this area that may in turn contribute to the onset of the ADHD-like phenotype. On this line, it may be inferred that SYN3 polymorphisms may lead to alterations of SN pr development, in turn promoting the onset of ADHD by affecting the establishment of proper GABAergic inhibitory afferents to this area.

Our data also indicate that Syn III-deficient primary mDN manifest early developmental alterations but a significant improvement of BDNF-dependent stimulation of dendritic branching and soma area, and disruption of Cdk5-mediated control of early dendritic arborization, similarly to or previous findings on primary cortical neurons [44]. This is reminiscent of studies on dopaminergic neurons derived from dental pulp stem cells from children with ADHD, which exhibit impairments in neurite development, but respond to BDNF treatment [69]. We also detected a decrease in both prepro-BDNF and mature BDNF in midbrain protein extracts from Syn III ko newborn mice that together with the BDNF hypersensitivity supports that Syn III deletion hinders BDNF production. Therefore, while Syn I acts downstream of BDNF signaling to regulate axonal growth [70], the very early expression of Syn III during neurodevelopment is pivotal to drive BDNF-mediated responses in the very early phases of development. Interestingly, although in this study we did not investigate whether *syn3* KD zebrafish larvae exhibited alterations in BDNF expression or signaling, it is known that BDNF is also expressed during very early phases of development in the brain of zebrafish embryos [71,72,73]. Therefore, we cannot exclude that the observed impairment in neurodevelopment in the *syn3* KD larvae, may be ascribed to BDNF alterations. Further studies are warranted to reliably validate this hypothesis.

Cdk5 plays a relevant role in BDNF-stimulated dendritic development [55] by mediating Syn III phosphorylation [44]. The here reported absence of roscovitine-mediated inhibition of dendritic intersections in Syn ko TH-positive midbrain primary neurons supports that Cdk5-mediated Syn III phosphorylation is relevant for the establishment of mDN dendritic arborization. Syn III polymorphisms may thus impinge on mDN development by affecting both BDNF production and BDNF/Cdk5-related dendritic mDN development. Remarkably, ADHD subjects exhibit reduced BDNF serum levels [74] and MPH, a drug which can bind to Syn III, can improve BDNF levels in ADHD patients [75,76]. In hypoxic-ischemic rats hippocampus, where Syn III is particularly abundant [77], MPH promotes BDNF production and neurogenesis [78,79], while chronic MPH treatment restores Cdk5 and TrkB levels in spontaneous hypertensive rats as a model of ADHD [80]. These findings, coupled to our evidence, strengthen that Syn III plays a key role in governing BDNF/Cdk5-mediated neuron development.

As BDNF expression can be regulated by neurotransmission [81,82,83,84], the Syn III-deletion-related neuronal activity alterations could be at the basis of the early BDNF deficiency in Syn III ko animals. However, Syn III controls axon development well before the first functional synapses are established [14]. Our primary cultures are produced from 13-day embryos, which already express BDNF without exhibiting mature synapses [85,86], which are not formed till 7 div [87,88,89,90]. As the BDNF-dependent overstimulation of Syn III ko mDN development was already present at 1 div, it is unlikely that Syn III ko-dependent alteration of synaptic activity may be at the basis of reduced BDNF production in the cell cultures. Despite this, since neuronal activity may have impacted on BDNF production in the brain of newborn mice, the reduction of BDNF in Syn III ko pups could also reflect a reduced neuronal function which appears in line with the observed neurodevelopmental impairment.

Finally, we also observed that the early and transient neurodevelopmental mDN impairment induced by Syn III deficiency are reproduced in human iPSCs-derived dopaminergic neurons, which exhibited a significant reduction of primary dendrites length, numbers, or soma size at 25 div that was fully recovered at 40 div.

## 5. Conclusions

Our observations indicate that Syn III is a key regulator of the early stages of vertebrate dopaminergic neurons development. Furthermore, they reveal that in mammals the Syn III-related control of mDN development involves BDNF/Cdk5 signaling. These findings own significant implications for deciphering the biological basis of ADHD and its therapeutic management.

## Figures and Tables

**Table 1:** List of the primary antibodies used in the WB studies and of their working dilutions.

Antibody	Manufacturer	Species	Dilution
Syn III	Synaptic System	Rabbit	1:2000
Syn II	Synaptic System	Rabbit	1:2000
Syn I	Synaptic System	Rabbit	1:2000
TH	Merck Millipore	Rabbit	1:2000
BDNF	abcam	Rabbit	1:1000
GAPDH	Sigma-Aldrich	Mouse	1:5000
α-Tubulin	Sigma-Aldrich	Mouse	1:2000

## Data Availability

The data presented in this study are available on request from the corresponding author. The data are not publicly available due to intellectual property restrictions.

## Supplementary Materials

The following supporting information can be downloaded at: https://www.mdpi.com/article/10.3390/cells11233902/s1. Figure S1: Syn III expression in Zebrafish and representative images of control NI Tg(*ngn1*:eGFP) and Tg(*neurod1*:eGFP) zebrafish embryos. Figure S2: Syn III identity and omology between species; Figure S3: Analysis of Syn III, II, Syn I levels in the nigrostriatal system of wt and Syn III ko mice; Figure S4: Behavioral analysis of 2 and 10-month-old wt and Syn III ko mice. Figure S5: WB-based analysis of BDNF levels in the SN of newborn Syn III ko and wt mice and representative images showing that Syn III ko mice mDN exhibited early delayed axonal growth; Figure S6: iPSCs-derived mDN showed early neurodevelopmental deficits; Video S1: Light sheet microscopy-based 3D imaging of the brain of Syn III-MO-injected Tg(*neurod1*:eGFP) zebrafish larvae 48 hpf; Video S2: Light sheet microscopy-based 3D imaging of the brain of ST-MO-injected Tg(*neurod1*:eGFP) zebrafish larvae 48 hpf; Video S3: Light sheet microscopy-based time-lapse imaging of the Syn III-MO-injected Tg(*neurod1*:eGFP) zebrafish larvae from 19 to 24 hpf; Video S4: Light sheet microscopy-based time-lapse imaging of the ST-MO-injected Tg(*neurod1*:eGFP) zebrafish larvae from 19 to 24 hpf; Video S5: Two-photon microscopy-based 3D visualization of the TH-positive neurons of the SN in the brain of Syn III ko newborn mice; Video S6: Two-photon microscopy-based 3D visualization of the TH-positive neurons of the SN in the brain of C57BL/6J wt newborn mice.
