# Supplementary material for: Synapsin III Regulates Dopaminergic Neuron Development in Vertebrates
Source: Cells. 2022 Dec 2;11(23):3902. doi: 10.3390/cells11233902 (PMC9739466; doi:10.3390/cells11233902)
Supplement: Supplementary file 1 [file cells-11-03902-s001.zip › Supplementary Materials-Figure.pdf]

Supplementary Figures

Figure S1

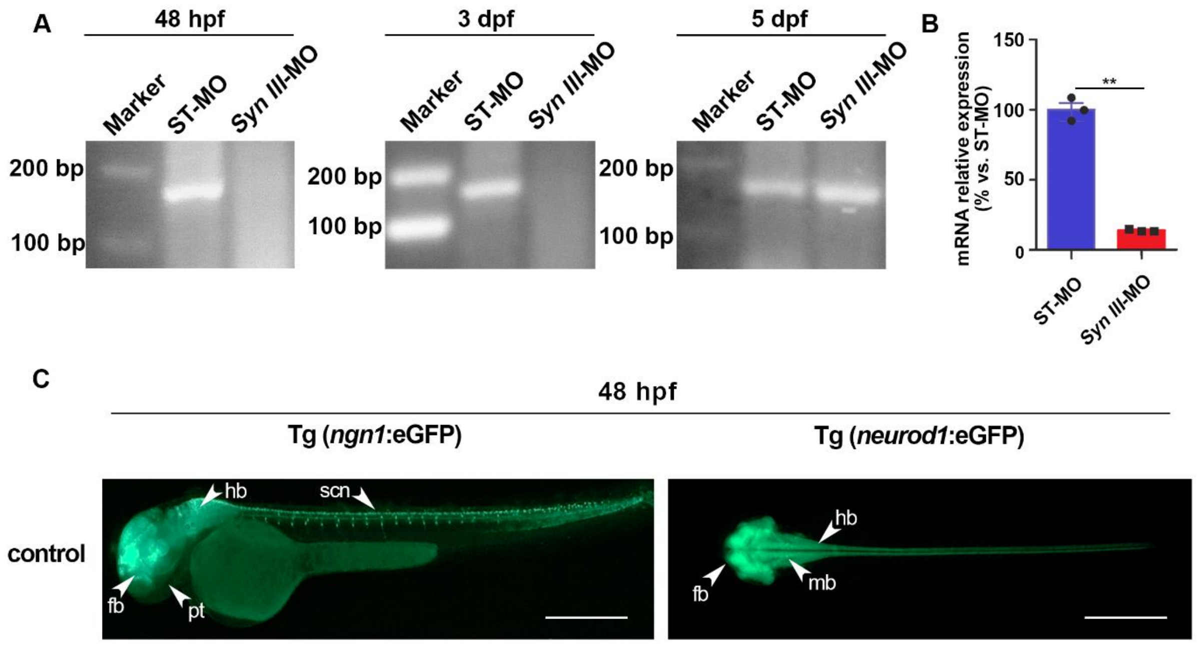

(A) Representative images showing Syn III mRNA RT-PCR products from 48 hpf, 3 dpf and 5 dpf pools of ST-MO and *Syn III*-MO embryos. (B) The graph shows Syn III qrt-PCR results of 48 hpf ST-MO and *Syn III*-MO embryos. Relative levels of Syn III mRNA were calculated using rpl13a as reference gene (** P<0.01 vs. ST-MO, Student's t-test).

(C) Representative lateral (left panels) and dorsal (right panels) views of *ngn1* and *neurod1* dependent eGFP fluorescence in the 48 hpf control Tg(*ngn1*:eGFP) and Tg(*neurod1*:eGFP) embryos, respectively. Scale bar Tg(*ngn1*:eGFP): 450 µm, Tg(*neurod1*:eGFP): 500 µm. Abbreviations: fb, forebrain; hb, hind brain; pt, posterior tuberculum; scn, spinal cord neurons; mb, midbrain.

Figure S2

A

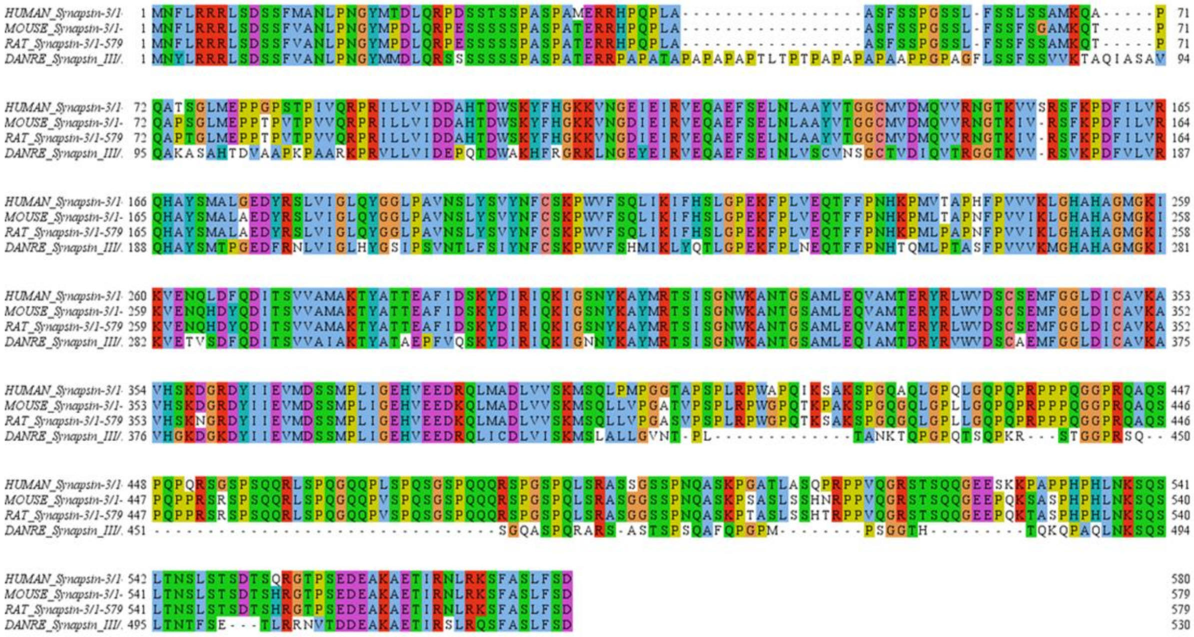

B

	Danio Rerio	Homo Sapiens	Mus Musculus	Rattus Norvegicus
**Danio Rerio**	*100%*	*67%*	*66%*	*66%*
**Homo Sapiens**		*100%*	*92%*	*92%*
**Mus Musculus**			*100%*	*98%*
**Rattus Norvegicus**				*100%*

C

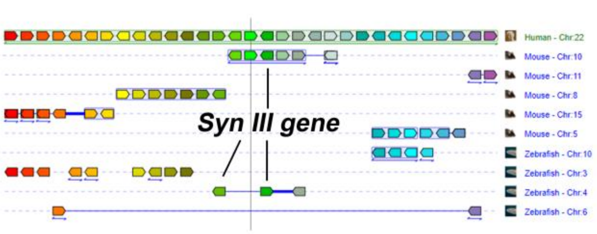

(A) Multiple alignments of mammalian and zebrafish Syn III protein. The amino acid sequences of *Homo Sapiens* SYN3 and *Danio Rerio syn3* were aligned using AlignX of Vector NTI Advance 10.1.1 based on the Clustal W algorithm. Subsequently, the alignment was edited with GeneDoc using the conservation mode for shading. Consensus residues were assigned based on the number of occurrences of the character in the column. Different colors were set to 100% conserved (blue shading), 80% or greater conserved (green shading), 60% or greater conserved (red shading), and less than 60% conserved (yellow, orange and white shading).

(B) Percentage of identity between *Homo Sapiens*, *Rattus Norvegicus*, *Mus Musculus* and *Danio Rerio* Syn III genes. The Figure shows the percentage of amino acid identity between mammalian and zebrafish Syn III gathered from multiple sequence alignment of Syn III proteins using ClustalW2 software.

(C) Gene trace synteny around the SYN3 locus between *Homo Sapiens*, *Mus Musculus* and *Danio Rerio* chromosome generated by Synteny Database (50-gene sliding window).

Figure S3

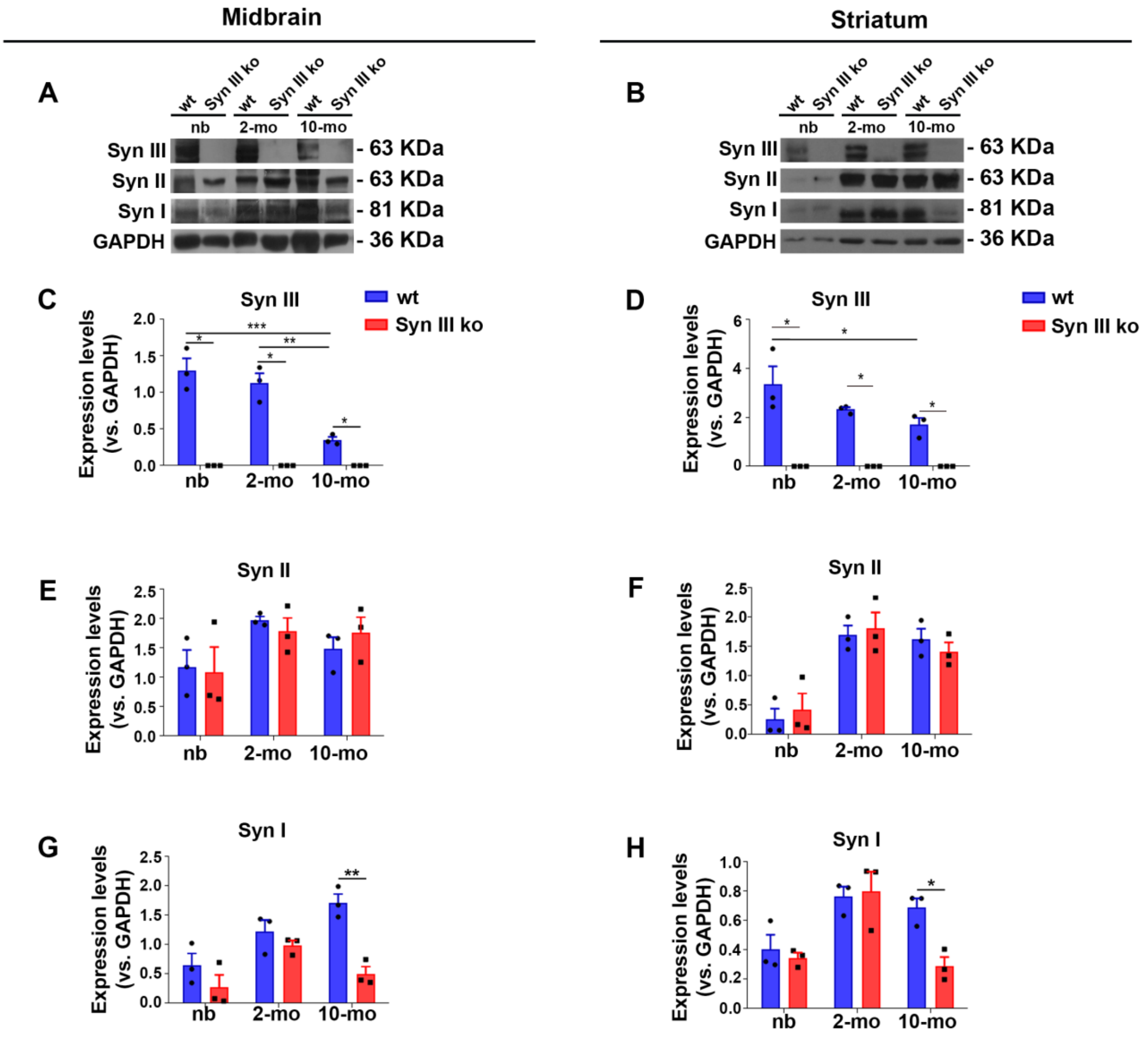

(A,B) Representative images showing Syn III, Syn II and Syn I immunopositive bands deriving from the WB analysis of midbrain and striatal protein extracts from newborn (nb), 2 or 10-month-old wt or Syn III ko mice.

(C,D) Absence of Syn III expression in Syn III ko mice. Data expressed as protein levels normalized vs GAPDH expression (* $p < 0.05$, ** $p < 0.01$, **** $p < 0.0001$, two-way ANOVA + Bonferroni's post-test, n = 3 animals *per* group).

(E,F) No difference in Syn II levels was observed at 0, 2 and 10 months of age in both areas. Data expressed as protein levels normalized vs GAPDH expression.

(G,H). Syn I levels were significantly decreased in 10-month-old Syn III ko mice when compared to wt littermates. Data expressed as protein levels normalized vs GAPDH expression (* $p < 0.05$, two-way ANOVA + Bonferroni's post-test, $n = 3$ animals *per* group).

Figure S4

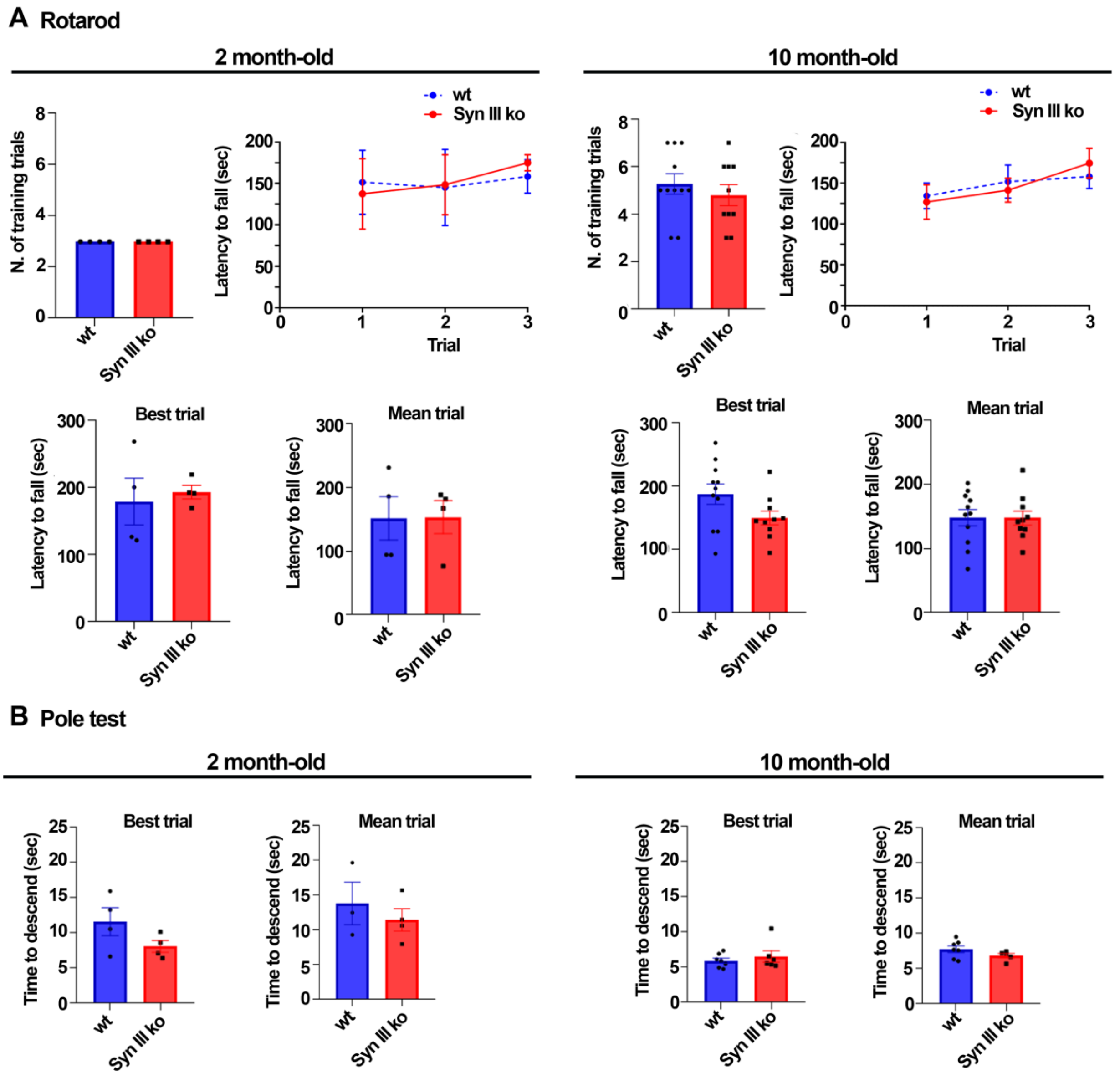

(A) Rotarod Test: graphs show the analysis of the number of training trials, the latency to fall of the best trial, the mean trial and the diagram of the latency to fall in each trial. No difference in these parameters was detected between wt and Syn III ko mice at 2 or 10 months of age (Student's t-test, n = 4-10 mice *per* group).

(B) Results of the pole test. No differences in the time to descend the pole was detected between the Syn III ko or wt mice in either the best or mean trial (Student's t-test, n = 4-6 mice *per* group).

Figure S5

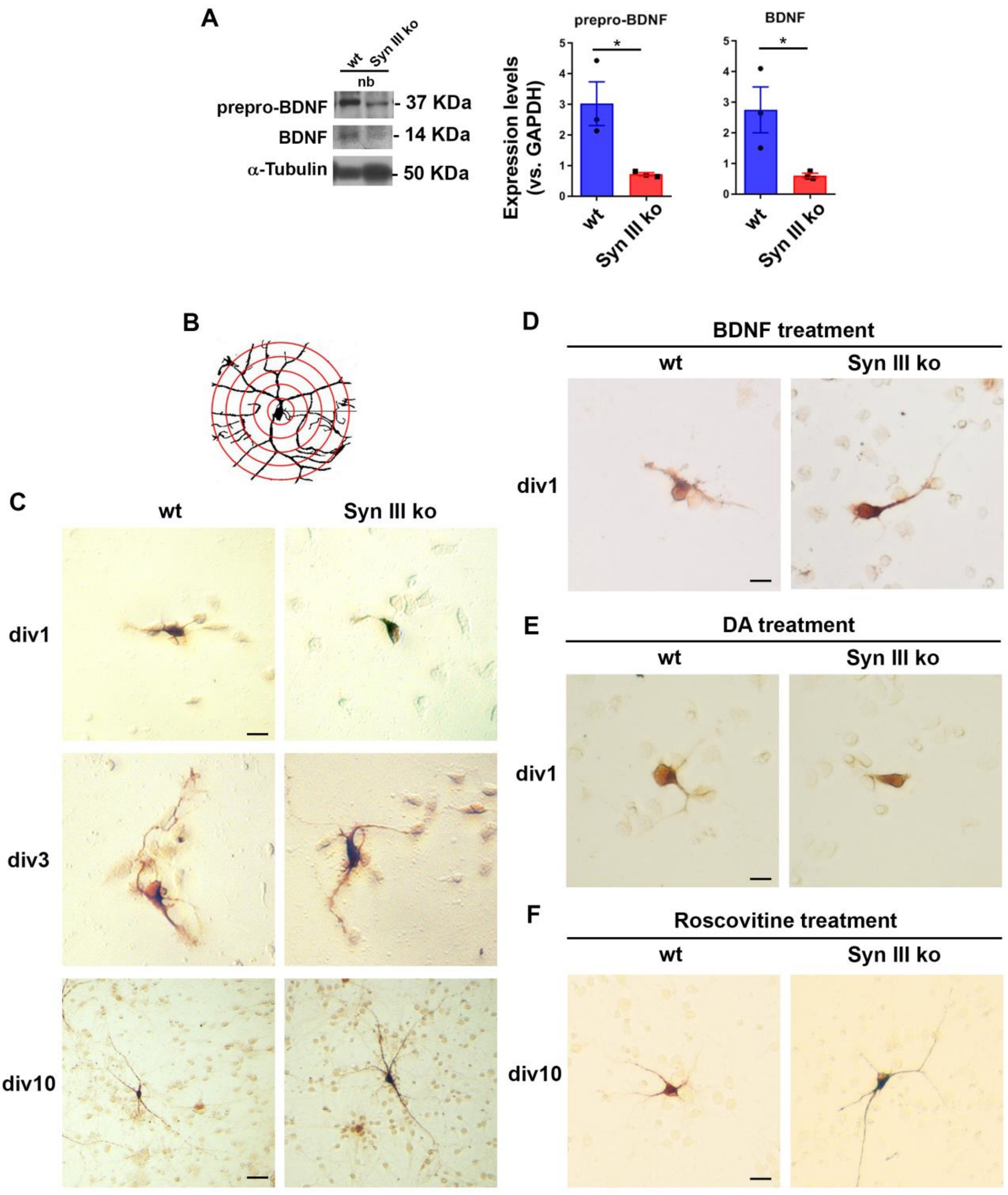

(A) Representative images and graphs showing prepro-BDNF and mature BDNF levels in the midbrain of newborn wt and Syn III ko mice. Please note the significant decrease of both prepro-BDNF and mature BDNF in the Syn III ko animals. Data are expressed as protein levels normalized

vs. α-tubulin expression (* $p < 0.05$, ** $p < 0.01$, *** $p < 0.001$; two-way ANOVA + Bonferroni's multiple comparisons test. n = 3 animals *per* each condition).

(B) Schematic representation of Sholl test analysis. The distance between concentric circle is fixed at 10 µm.

(C) Representative images of mDN from wt and Syn III ko mice at div 1, 2, 10. Scale bars: 10 µm for 2 and 10 div; 20 m for 10 div.

(D) Representative images of mDN from wt and Syn III ko mice at div 1 receiving BDNF treatment. Scale bar, 10 µm.

(E) Representative images of mDN from wt and Syn III ko mice at div 1 receiving DA treatment. Scale bar, 10 µm.

(F) Representative images of mDN from wt and Syn III ko mice at div 10 receiving roscovitine treatment. Scale bar, 10 µm.

Figure S6

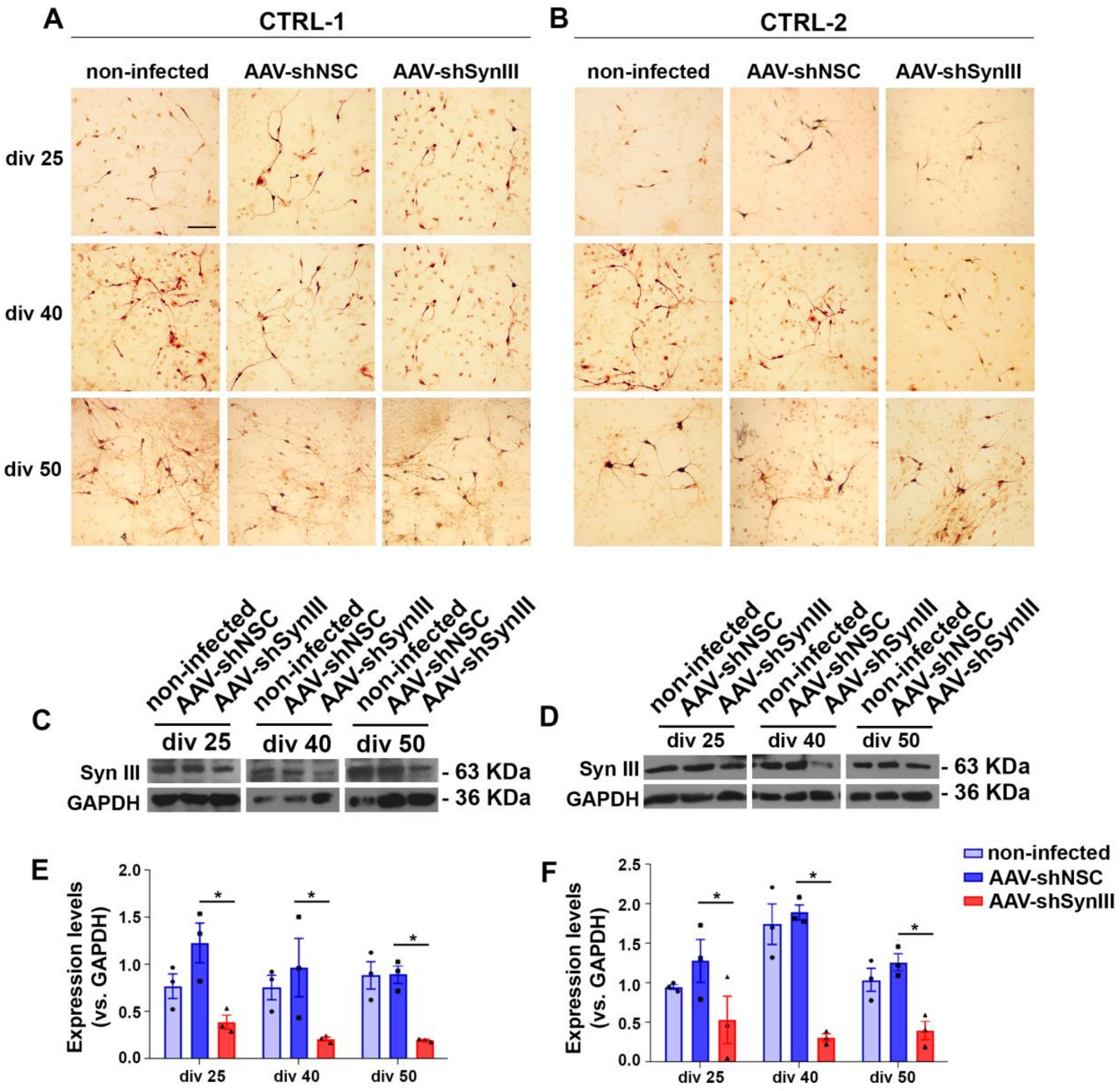

(A-B) Representative images of mDN from CTRL-1 (A) and CTRL-2 (B)-derived iPSCs at div 25, 40, 50. Scale bar 100 µm.

(C-F) Representative western blot results showing the reduced Syn III expression at div 25, 40 and 50 in AAV-shSynIII infected neurons of CTRL-1 and CTRL-2 when compared to AAV-shNSC-infected neurons (* $p < 0.05$, two-way ANOVA + Bonferroni's multiple comparisons test. n = 3 replicates *per* each experimental condition).
